# The oncogenic fusion landscape in pediatric CNS neoplasms

**DOI:** 10.1007/s00401-022-02405-8

**Published:** 2022-02-15

**Authors:** Mieke Roosen, Zelda Odé, Jens Bunt, Marcel Kool

**Affiliations:** 1 Princess Máxima Center for Pediatric Oncology, 3584CS Utrecht, The Netherlands; 2 Hopp Children’s Cancer Center (KiTZ), 69120 Heidelberg, Germany; 3 Division of Pediatric Neurooncology, German Cancer Research Center DKFZ and German Cancer Consortium DKTK, 69120 Heidelberg, Germany

**Keywords:** Pediatric CNS tumors, Oncogenic fusion protein, Kinase, Transcription factor, Brain tumor

## Abstract

Pediatric neoplasms in the central nervous system (CNS) are the leading cause of cancer-related deaths in children. Recent developments in molecular analyses have greatly contributed to a more accurate diagnosis and risk stratification of CNS tumors. Additionally, sequencing studies have identified various, often entity specific, tumor-driving events. In contrast to adult tumors, which often harbor multiple mutated oncogenic drivers, the number of mutated genes in pediatric cancers is much lower and many tumors can have a single oncogenic driver. Moreover, in children, much more than in adults, fusion proteins play an important role in driving tumorigenesis, and many different fusions have been identified as potential driver events in pediatric CNS neoplasms. However, a comprehensive overview of all the different reported oncogenic fusion proteins in pediatric CNS neoplasms is still lacking. A better understanding of the fusion proteins detected in these tumors and of the molecular mechanisms how these proteins drive tumorigenesis, could improve diagnosis and further benefit translational research into targeted therapies necessary to treat these distinct entities. In this review, we discuss the different oncogenic fusions reported in pediatric CNS neoplasms and their structure to create an overview of the variety of oncogenic fusion proteins to date, the tumor entities they occur in and their proposed mode of action.

**Supplementary Information:**

The online version contains supplementary material available at 10.1007/s00401-022-02405-8.

## Introduction

Neoplasms in the central nervous system (CNS) account for the second most common cancer and are the leading cause of cancer related deaths in children [165]. However, overall survival and therapy response vary widely between and within different pediatric CNS tumor entities. Treatment options include resection followed by radio- and/or chemotherapy depending on the patient’s age. This intensive therapy often has detrimental and long-term side effects, while the overall survival remains low for many entities or subentities. This is partly due to the lack of molecularly stratified trials and entity specific treatments [162].

Pediatric CNS tumors comprise a vast and expanding spectrum of molecularly defined entities [137]. Histologically, these entities present with various morphological patterns that are not unique for a single entity, thus complicating an accurate diagnosis based on standard histopathology alone. The need for a proper diagnosis together with the need for adequate targeted treatment options calls for identification of specific driver events and genetic and molecular signatures that better define tumor types. Molecular characterization by means of next generation sequencing (NGS) has added to the better understanding of these tumor signatures and has emphasized the oncogenic differences between pediatric and adult cancers. While adult neoplasms have high numbers of somatic mutations, these often lack in pediatric neoplasms [236]. In contrast, pediatric tumors show a higher frequency of germline alterations, copy number alterations, and structural alterations such as enhancer hijacking events and gene fusions as possible oncogenic drivers [4]. While already being well-described in pediatric hematological neoplasms and sarcomas, fusion proteins are now also emerging as important oncogenic driver events in pediatric CNS neoplasms [7, 98, 109, 144, 147, 150, 176].

## Fusion genes in neoplasms

The first oncogenic fusion gene was already detected in the 1980s in chronic myeloid leukemia, but it was not until the use of NGS technologies for detection of rearrangements in the cancer transcriptome that the majority of fusion genes has been detected [73, 114, 191, 203]. The chromosomal rearrangements causing these fusions can be either balanced or unbalanced. An unbalanced rearrangement is often the result of the deletion of part of a chromosome, which then combines two genes that were formerly separated by an interstitial chromosomal segment. Balanced rearrangements can occur due to translocations, insertions, or inversions of chromosomal parts. Both balanced and unbalanced aberrations can occur between genes on the same chromosome (intra-chromosomal) as well as between genes on separate chromosomes (inter-chromosomal). These different types of rearrangements have been extensively reviewed [148, 149].

The outcome of these rearrangements is either an altered expression of one of the gene products, or a new fusion product due to the combination of the transcripts of both genes. The first occurs when the coding sequence of one gene is placed next to the promoter sequence of another gene. Although this is not a fusion protein, it does lead to altered expression levels and is seen in, for example, medulloblastoma (e.g., *DDX31*–*GFI1B*), neuroblastoma (e.g., *HAND2*–*MYC*), multiple myeloma (e.g., *PRDM1*–*MYC*), Burkitt lymphoma (e.g., *IG*–*MYC*), embryonal tumor with multilayered rosettes (*TTYH1*–*C19MC*) and in the newly identified CNS neuroblastoma tumors with *FOXR2* activation (e.g., *JMJD1C*–*FOXR2*) [2, 109, 134, 161, 216, 253]. Such type of rearrangements are now known as enhancer hijacking events and will not be discussed in this review [161]. A new fusion product occurs when the promotor and 5′-coding region of one gene is fused with the 3′ coding region and UTR of the second gene, resulting in a chimeric transcript that can be translated into a fusion protein. Although many of these fusions have been identified on a RNA level, in this review we will address them as fusion proteins since their functional domains and protein functions are being discussed.

For this review, we present an overview of all chimeric proteins in pediatric CNS neoplasms that are either identified in two or more independent studies or for which additional molecular validation has been presented.

## Chimeric proteins in pediatric CNS tumors

**Fig. 1:**
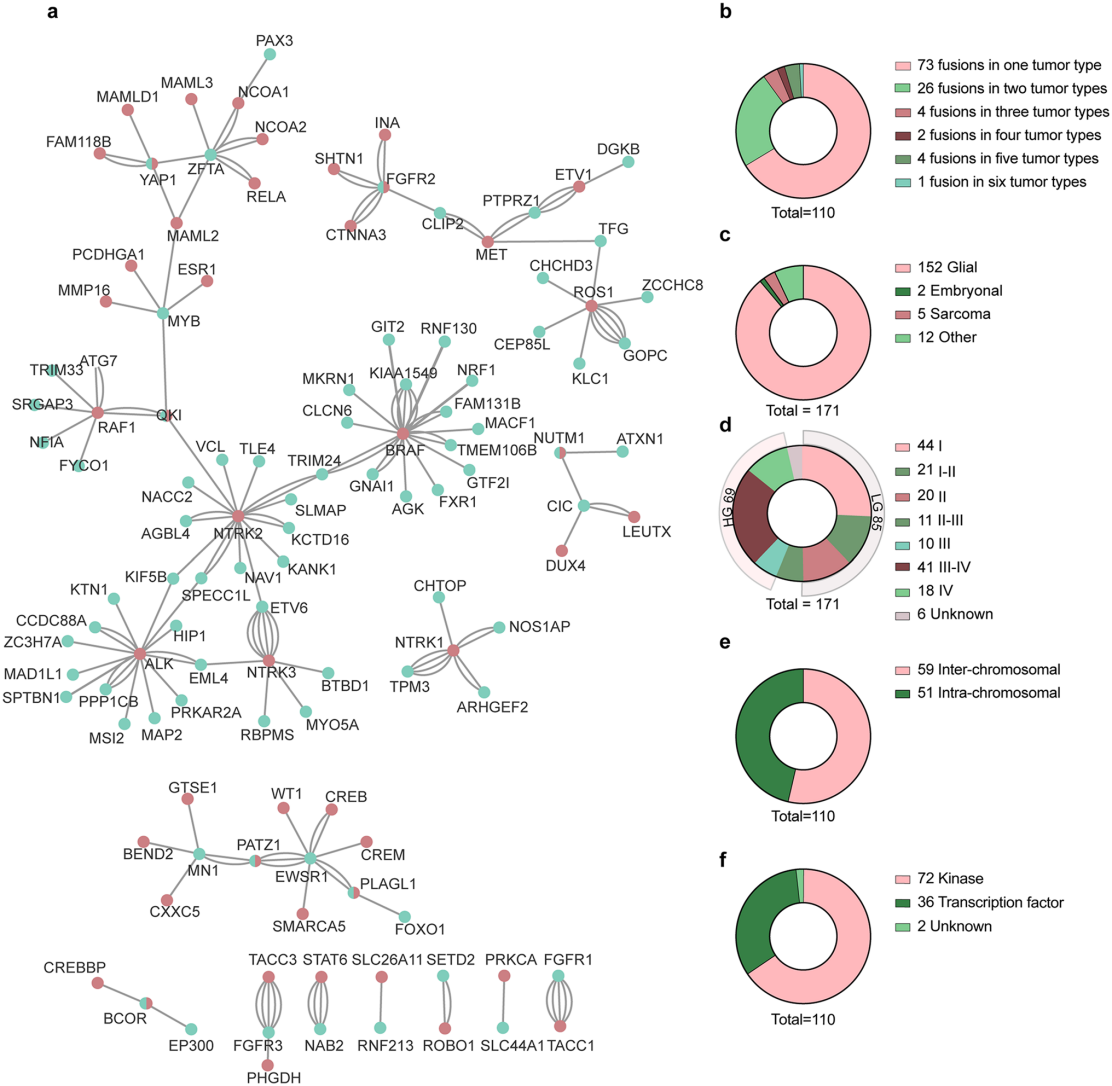
Characteristics of fusion proteins observed in pediatric brain tumors. **a** Complete fusion partner network in pediatric CNS neoplasms. Green = N terminal partner, red = C terminal partner. Multiple connecting lines indicate that fusions were identified in multiple tumor types. **b** The majority (73/110) of fusions have been observed in a single tumor type. 37/110 fusions have been detected in two or more tumor types. The tumor types are annotated as mentioned in the original publication, it might be possible that some tumor types have been wrongly diagnosed or that diagnosis was changed after this publication. This is not considered. **c** The majority (152/171) of the pediatric CNS tumors with fusions present are glial tumors. **d** Based on WHO classification there are slightly more pediatric CNS low grade (LG) tumors than high grade (HG) tumors with driving fusion proteins. The WHO classification is based on the original publication and/or the tumor type. **e** Fusions in pediatric CNS tumors are slightly more often the results of inter-chromosomal rearrangements than intra-chromosomal rearrangements. **f** Most of the fusion proteins have at least one partner that functions as a kinase. Most other fusions have at least one transcription factor

Fusion proteins have been reported in 43 different pediatric CNS neoplasms. By totalling all fusions per entity, 171 distinct fusion–entity combinations have been detected (Fig. 1a. Online Resource) [3, 8, 11, 13–15, 17, 19, 21–23, 27, 28, 30, 33, 34, 36, 39, 40, 42, 44, 46, 47, 50, 58, 59, 62–64, 66, 69, 70, 74, 76, 79, 81–87, 93, 96, 97, 99, 101, 105–107, 111, 114–116, 119, 121, 127–129, 132, 133, 135, 142, 145, 146, 154, 155, 157, 159, 162, 164, 167, 169, 175–177, 180–182, 184, 189, 190, 192, 198, 201, 202, 204–207, 210, 214, 216, 217, 221, 222, 225–229, 235, 239, 240, 242, 244–246, 248–250, 252, 255]. These 171 combinations exist of 110 unique 5’ and 3’ fusion partner gene combinations. The majority (66%; 73/110) of these unique fusions are entity specific (Fig. 1b), and their detection could aid diagnosis. For example, Yes1 associated transcriptional regulator (YAP1)–MAMLD1 fusions are restricted to supratentorial ependymoma (ST-EPN). The remaining 37 (34%) fusion proteins have been detected in multiple (two to six) tumor types (Fig. 1a, b)*.* Additionally, there are genes with multiple fusion partners such as neurotrophic receptor tyrosine kinases (NTRKs) and fibroblast growth factor receptors (FGFRs). However, the fusions with one of the partner genes may still be specific to a tumor type, while other fusion partners occur in multiple tumor types. With the ever-expanding sequencing of tumors, these unique fusion genes might be detected in the future in other tumor types as well. Furthermore, fusions that we have not included here due to their presence in only a single case might in the future be confirmed in other cases adding to the complexity of the fusion network in pediatric CNS tumors.

Strikingly, most of the reported fusion proteins (89%; 152/171) are observed in glial tumors, while only five (3%) fusions are present in sarcomas, two (1%) in embryonal tumors and 12 (7%) fusions occur in other CNS tumor diagnoses (Fig. 1c, Online Resource 1). Most of the well-known fusion proteins, such as KIAA1549–BRAF, are especially prevalent in pediatric low-grade gliomas (LGG). Thus we examined whether the 171 identified fusion–entity pairs represent more low-grade tumors, based on data extracted from the original reports where tumor types were graded according to the WHO grading system [136]. When we divide tumors into low grade (I–II) and high grade (III–IV), low-grade tumors (50%; 85/171) harbor more fusions than high-grade tumors (40%; 69/171) (Fig. 1d). Since prevalence is not taken into account, it might be possible that overall, fusions are even more common in a certain tumor grade. For example, KIAA1549–BRAF has been detected in at least five different entities, however this fusion is reported in 70–80% of the pilocytic astrocytomas, making the fusion the most prevalent in grade I tumors [91, 112]. More than half of the reported unique fusions are inter-chromosomal (54%; 59/110) (Fig. 1e). However, due to the difficulties in detecting intra-chromosomal fusions, their incidence number might be underrepresented and might therefore increase with improved detection algorithms.

**Fig. 2:**
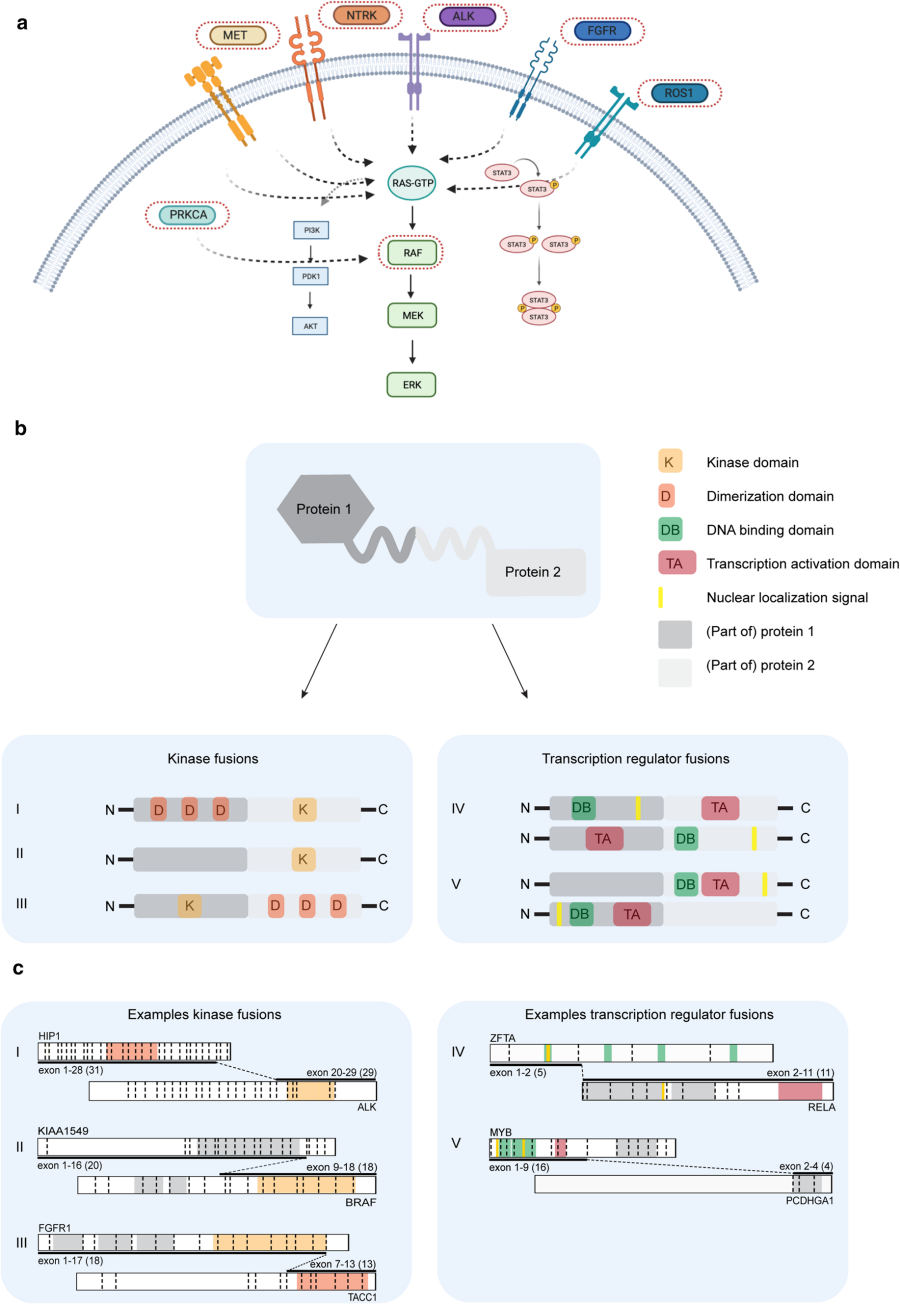
Fusion proteins are most common in the RAS/MAPK pathway. **a** Schematic representation of the RAS/MAPK, PI3K/AKT/mTOR and JAK/STAT pathway. Kinases implicated in fusions in pediatric CNS tumors are marked with a red dashed circle. Created with BioRender.com **b** Five common trends in fusion proteins observed in pediatric CNS tumors. I. C-terminal kinase protein fused to a protein with dimerization domains. II. C-terminal kinase protein fused to a protein that is highly expressed in the CNS. III. N-terminal kinase protein fused to a protein with dimerization domains. IV. Transcription factors with a transcription activation domain fused to proteins with a DNA binding domain. V. C-terminal transcription factors with a transcription activation domain and a DNA binding domain fused to a protein with no clear functional domains. **c** Examples of oncogenic fusions in pediatric CNS tumors that belong to the five different fusions types as mentioned in **b**. For every protein, the exons that are retained in the fusion protein are specified, as well as the total exons (in between brackets) in the original protein

**Fig. 3:**
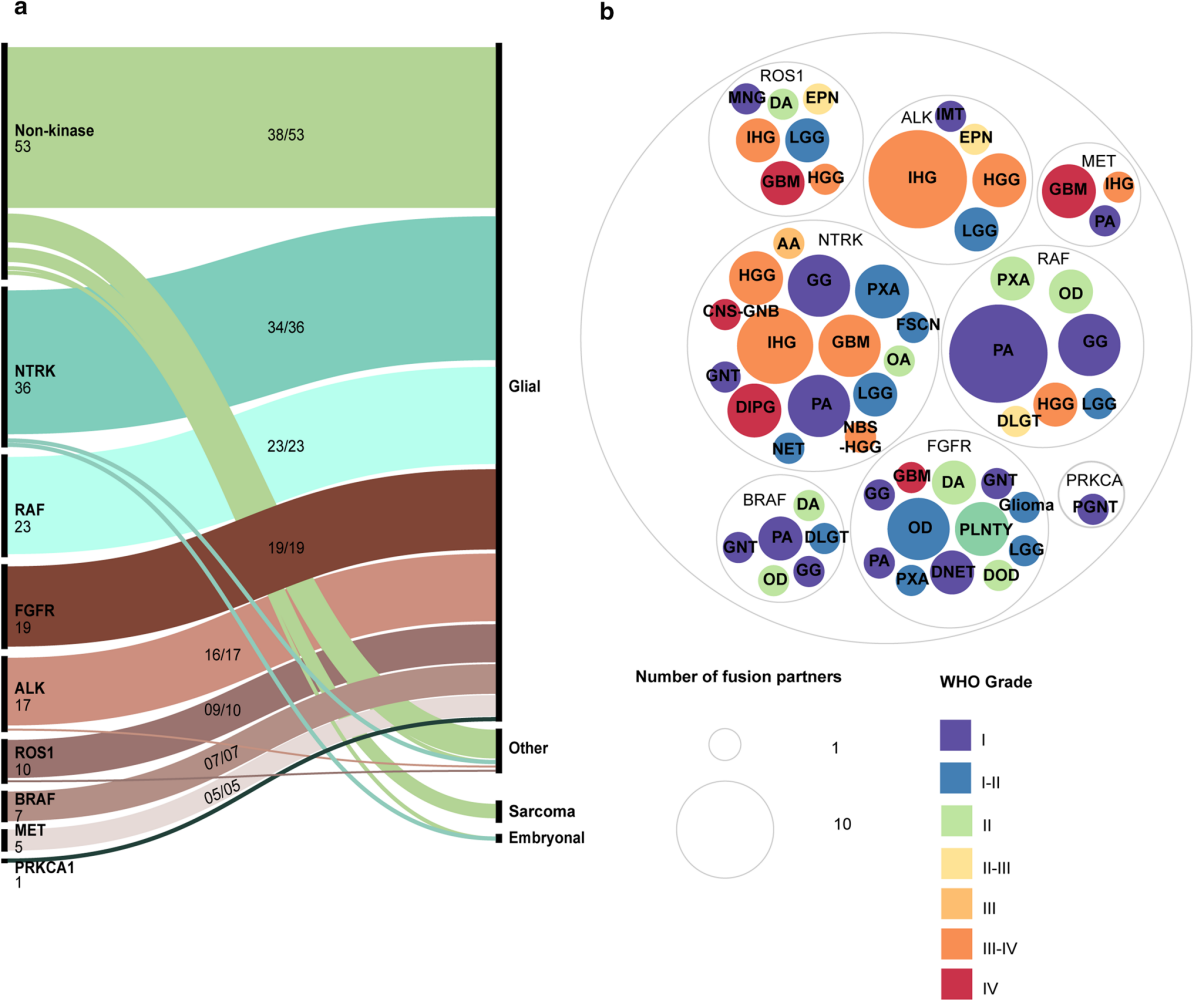
Characteristics of kinase fusions in pediatric CNS neoplasms **a** Eight kinase families are responsible for all kinase fusions that are mainly present in glial tumors. **b** Kinase proteins are not limited to one pediatric CNS tumor type but are present in multiple different tumor types. Graphs made by https://app.rawgraphs.io. *AA* anaplastic astrocytoma, *DA* diffuse astrocytoma, *DIPG* diffuse intrinsic pontine glioma, *DLGT* diffuse leptomeningeal glioneural tumor, *DNET* dysembryoplastic neuroepithelial tumor, *DOD* diffuse oligodendroglioma, *EPN* ependymoma, *FSCN* fibroblastic spindle cell neoplasm, *GBM* glioblastoma, *GNT* glial neuronal tumor, *CNS-GNB* central nervous system ganglioneuroblastoma, *HGG* high grade glioma, *IHG* interhemiscpheric glioma, *IMT* inflammatory myofibroblastic tumor nervous system—ganglioneuroblastoma, *LGG* low grade glioma, *MNG* meningioma, *NBS-HGG* non brain stem high grade glioma, *NET* neural epithelial tumor, *OA* oligoastrocytoma, *OD* oligodendroglioma, *PA* pilocytic astrocytoma, *GG* ganglioglioma, *PGNT* papillary glial neuronal tumor, *PLNTY *polymorphous neuro-epithelial tumor of the young, *PXA* pleomorphic xanthoastrocytoma

For most of the unique fusion proteins (65%; 72/110), one partner is either a tyrosine or a serine/threonine kinase (Fig. 1f). Interestingly, these 72 fusions are comprised of 12 different kinase genes, classified into seven kinase families: (1) NTRK family (NTRK1–3), (2) Raf proto-oncogene, serine/threonine kinase (RAF) family (RAF1/BRAF), (3) FGFR family (FGFR1–3), (4) ALK receptor tyrosine kinase (ALK), (5) ROS proto-oncogene 1, receptor tyrosine kinase (ROS1), (6) MET proto-oncogene, receptor tyrosine kinase (MET), and (7) protein kinase C alpha (PRKCA) (Fig. 2a). PRKCA only accounts for one fusion–entity pair and will thus not be discussed further. In pediatric CNS neoplasms these 72 kinase fusions appear as 118 unique fusion–entity pairs, mainly in glial entities (97%; 114/118), except for some NTRK- (2%; 2/118), ALK- (1%; 1/118) and ROS1- (1%; 1/118) fusions that have been identified in non-glial tumors (Fig. 3a) [133, 145, 190].

We determined whether the different groups of kinase fusions have a preference towards a certain tumor type. Most are present in multiple different tumor types (Fig. 3b). However, ALK fusions mostly occur in infant hemispheric gliomas (IHG), while pilocytic astrocytomas are mainly characterized by BRAF and RAF1 fusions. Furthermore, no BRAF and RAF1 fusions were found in glioblastoma, which are characterized by fusions with NTRK, FGFR, MET or ROS1. In oligodendrogliomas there are mostly fusions with members of the FGFR family. Nonetheless, it should be kept in mind that there may be discrepancies due to updated nomenclature and differences in diagnostic methods and criteria used.

The 39 fusions lacking a kinase domain, representing 53 fusion–entity pairs, are mainly observed in glial tumors (72%; 38/53) (Fig. 3a). However, they also occur in embryonal tumors (2%; 1/53), sarcomas (9%; 5/53) and other CNS tumor diagnoses (17%; 9/53). They cover a variety of fusion partners, including recurrent partners such as EWS RNA binding protein 1 (EWSR1), MYB proto-oncogene, transcription factor (MYB), and zinc finger translocation associated (*ZFTA*, formerly *C11orf95*) [15, 27, 33, 105, 132, 176, 207, 226, 245, 255]. Instead of harboring kinase domains, these fusions are mostly characterized by transcription factors and partners with a DNA binding domain, indicating that besides kinases, these are important partners in oncogenic fusions in pediatric CNS tumors.

## Receptor Tyrosine Kinases and the MAPK pathway in CNS tumors

Ten of the 13 kinase families involved in fusion proteins are receptor tyrosine kinases (RTKs), while BRAF, RAF1 and PRKCA serine/threonine kinases (STKs) are part of their downstream pathways (Fig. 2a). Under physiological conditions, RTKs, only activate the downstream pathways upon binding of extracellular growth factors, thereby inducing proliferation, differentiation, and cell survival [20, 95]. In pediatric CNS neoplasms, mutations or fusions cause dimerization and cross-phosphorylation of the intracellular domain independent of this mitogenic signal. Therefore, the RTKs constitutively activate their downstream signaling pathways, including the RAS/MAPK pathway. BRAF, RAF1 and PRKCA are all signaling through this pathway. During development, the RAS/MAPK pathway is important in cortex, midbrain and cerebellum formation [194, 197]. Its role in neurogenesis is especially interesting in regards to glial pathogenesis, considering that the cell-of-origin for gliomas is now proposed to be a neural stem cell or neural precursor instead of a post-mitotic glial cell [96, 130]. The exact mechanism how the RAS/MAPK pathway contributes to brain development is still controversial and potentially also depends on the spatial localization of the cell [25, 68]. Nonetheless, the RAS/MAPK pathway is often deregulated in gliomas [90, 91, 96, 98, 113, 128]. High-grade gliomas as well as low-grade gliomas are defined by aberrations in the pathway, although the aberrations differ between different entities. High-grade gliomas more often have aberrations in the upstream components such as the receptor tyrosine kinase NTRK, while the low-grade gliomas such as pilocytic astrocytoma have more aberrations in the intracellular RAF kinases [90]. Genomic studies showed that almost all of the pilocytic astrocytomas bear an aberration in the RAS/MAPK pathway without additional mutations or alterations [97, 179, 245].

## Kinase fusions types

We have distinguished three different types of kinase fusion proteins. In the first type, the kinase domain of the RTK/STK is always retained. The RTK/STK is the C-terminal partner, meaning that the expression of the chimeric protein is driven by the promoter of its N-terminal fusion partner (Fig. 2b I). This may lead to an elevated RTK/STK expression level compared to the endogenous RTK/STK expression, as is seen in fusions with CLIP2, EML4, ETV6, KIF5B, QKI, and TFG. Due to these genes being relatively high expressed in the glial cell lineages, the oncogenic C-terminal RTK/STK expression is elevated. These fusion partners further contribute to the oncogenicity of the fusion protein by facilitating the dimerization of the RTK in absence of its ligands, an example of such fusion is HIP1–ALK (Fig. 2c). In most of these kinase fusions type I (Fig. 2b) the extracellular domain as well as the transmembrane domain of the RTK is lost and therefore the fusion protein localizes to the cytoplasm. Since the RAS signaling is lipid membrane-dependent it remains to be elucidated how oncogenic fusions activate the RAS/MAPK pathway [126]. Some of these chimeric proteins, such as EML4–ALK, can induce lipid membrane-independent RAS signaling by formation of protein-based granules [231]. Further research is necessary to show if more fusion proteins make use of this mechanism.

BRAF fusions belong to the second type of fusions, where the kinase domain is retained in the C-terminal partner but there is no common oncogenic contribution within the N-terminal partners (Fig. 2b II, c II). In FGFR fusions, who belong to the third type of fusions, FGFR retains its kinase domain and is always the N-terminal partner. The C-terminal partner almost always contains a dimerization domain, as also seen with TACC1 (Fig. 2c III). Since FGFR is the N-terminal partner it does not depend on the expression level of their partner (Fig. 2b III). A reason for this could be that tumors with FGFR fusions arise during brain development at a stage that *FGFR* genes are highly expressed in progenitors, or alternatively, because the regulatory domains in the 3′ UTRs of *FGFR* genes that negatively regulate *FGFR* expression are lost in these fusions [38].

## Fusions involving kinases in pediatric CNS tumors

### RAF fusions

BRAF and RAF1 are both RAF kinases that occur as fusion proteins with various and different partners. BRAF is seen as an oncogenic driver in a wide variety of solid and hematological malignancies. Most of the aberrations found in BRAF are mutations, and by far the most common mutation occurs within the kinase domain at amino acid V600 (V600E). While the V600E mutation in melanoma is approved for therapy with BRAF inhibitors dabrafenib and vemurafenib, these same inhibitors are controversial in other malignancies and they seem not able to inhibit tumorigenesis in pediatric astrocytomas harboring BRAF fusions and may even lead to tumor progression [103]. However, the MEK inhibitor selumetinib has recently been tested in pediatric patients with LGG harboring the well-known KIAA1549–BRAF fusion and has shown to be effective in a phase I and II trial [16, 55, 56]. Many new BRAF fusions have been identified over the last couple of years, with one study identifying 29 different BRAF fusions across seven different tumor types [189]. In pediatric CNS tumors, 14 different BRAF fusions have been described (Online Resource 2). In these fusions, the C-terminal part of BRAF is fused to the N-terminal part of its partner. The most common partner is KIAA1549 but other N-terminal partners like CLCN6, GNAI1, GTF2I, GIT2, or FAM131B, have also been reported [76, 97, 184, 192, 225, 245]. The BRAF kinase domain (encoded by exon 11–18) is retained in all pediatric CNS fusions. Most fusion breakpoints occur at the 9th exon of BRAF and in all fusions the inhibitory regulatory domain that is located within the first six exons is cut off by the fusion. So far there has been no evidence that the N-terminal partner is of specific importance other than the removal of the regulatory domain [110, 208, 238]. Six of the 14 fusion partners have either no significantly important domain in the fusion part or a domain of unknown function. The other fusion partners have domains that vary from E3 ligase, zinc-finger to coiled-coil, thus showing no clear trend in the N-terminal fusion partners.

RAF1 is the second member of the RAF kinase family that is implicated in fusions and functions alongside BRAF in the RAS/MAPK pathway. RAF1 fusions are identified in pediatric LGG (p-LGG) as well as several adult malignancies such as prostate cancer, breast cancer, pancreatic cancer and thyroid cancer [171, 215, 243]. As with BRAF fusions, due to the limited number of discovered fusion events, their prevalence and oncogenic potential as well as the effectivity of inhibitory compounds is still being elucidated. While second generation RAF inhibitors are effective for BRAF fusions, they are not effective for RAF1 fusions. Vemurafenib has even been found ineffective targeting RAF1 fusions in pediatric astrocytoma due to a paradoxical activation of the RAS/MAPK pathway [208]. RAF1 has at least six different fusion partners in pediatric CNS tumors (Online Resource 3). It is hypothesized that the N-terminal fusion partners in RAF1 fusions are important for the oncogenic potential of the fusion proteins in contrast to the partners of BRAF. Moreover, several partners such as QKI and SGRAP have already been implicated in other malignancies as well [15, 37, 118, 123]. Furthermore, all RAF1 fusion proteins identified in pediatric CNS tumors have N-terminal partners that possess a coiled-coil or other dimerization domain, meaning they belong to type I of kinase fusions (Fig. 2b). This might indicate that the dimerization of these fusion partners is necessary for the oncogenic mechanism of the fusion protein. Subsequently, RAF inhibitors may not be able to disrupt these oncogenic dimers and are thus unable to inhibit downstream signaling. For further research, combination therapies should be considered that combine a RAF inhibitor together with molecules that block the dimerization of N-terminal fusion partners.

### ALK fusions

ALK belongs to the insulin receptor superfamily of RTKs. Under physiological conditions, the gene translates into a membrane bound receptor in nerve cells where it activates next to MAPK also the PI3K/AKT/mTOR and JAK/STAT pathways [75]. ALK rearrangements are common in all types of adult and pediatric cancers. Over 30 fusions have been described in various tumor types [35]. In pediatric CNS tumors, 13 different partners have been observed (Online Resource 4). These fusions are mainly found in IHGs and include HIP1–ALK, EML4–ALK and PPP1CB–ALK as well as the more recently identified ZC3H7A–ALK, MAD1L1–ALK and MSI2–ALK [41]. All ALK fusions contain the complete kinase domain of ALK at the C-terminal end, while the N-terminal partners retain variable domains in the chimeric protein, although most of the N-terminal partners have a coiled-coil or dimerization domain (69%; 9/13). ALK fusions are therefore part of the type I kinase fusions (Fig. 2b). The extent of oligomerization that is endorsed by these domains differs per partner, leading to a diversity in the oncogenic potential of the different ALK fusions [212, 213]. Interestingly, due to these variables, different ALK-fusion positive tumors have varying sensitivity to ALK inhibitors such as crizotinib, ceritinib, alectinib, brigatinib, and lorlatinib. Not only the expression, dimerization and stability of the chimeric protein plays a role in this, but also the signaling pathway that is activated, should be considered when treating patients with ALK inhibitors. Fusion proteins that induce a > 0.5 ratio phosphorylated ALK/total ALK can activate the MAPK pathway [35]. Other fusions potentially activate different signaling pathways and this influences the oncogenic mechanism and its sensitivity to the different ALK inhibitors. In vitro and in vivo tests with ALK inhibitors showed pre-clinical evidence for tumor reduction in a PPP1CB–ALK positive tumor as a response to ALK targeted therapy with lorlatinib [40]. However, the effect on tumors with different ALK fusions remains unknown.

### ROS1 fusions

ROS1 closely resembles ALK both in sequence and structure. Both RTKs have an extracellular domain, a transmembrane domain and an intracellular kinase domain and both receptors signal via the RAS/MAPK as well as the JAK/STAT and PI3K/AKT/mTOR pathways [1, 160, 172, 254]. ROS1 fusions are identified in several tumor types and are relatively common in non-small cell lung cancer (NSCLC), spitzoid neoplasms and inflammatory myofibroblastic tumors [170, 232]. In pediatric CNS tumors six different fusions have been described (Online Resource 5). In contrast to ALK, the most common ROS1 fusion, GOPC–ROS1, induces oncogenic signaling by translocating to the Golgi apparatus rather than by dimerization [29]. However, more recent research shows that dimerization and kinase activation is also a key step in the constitutive activation of ROS1 in fusion proteins [31]. The exact oncogenic mechanism of the chimeric protein is also determined by the fusion partner [100]. Although the breakpoints might be slightly different, all fusions retain the kinase domain of ROS1 encoded by exons 36–41 [220, 233]. Furthermore, all fusions lose the transmembrane domain of ROS1, leading to a cytoplasmic location of the fusion protein. Additionally, all the fusion partners contain a coiled-coil domain and sometimes an additional leucine zipper domain that leads to the dimerization of the fusion protein and hence the ligand independent activation of the ROS1 kinase. Although this type I kinase fusion activation is the most credible reason for the oncogenic signaling to date, it is possible that the loss of N-terminal regulatory domains as in type II fusions also play a role in enhanced signaling of ROS1 (Fig. 2b). More research is required to determine whether other mechanisms also play a role in ROS1 kinase activation [232]. Specific ROS1 inhibitors do not exist but there is evidence that next generation tyrosine kinase inhibitors (TKI) like entrectinib are potent against ROS1 fusions [53]. Furthermore, entrectinib unlike other TKI can sustain prolonged CNS exposure making it a suitable drug for treating ROS1 positive primary CNS tumors [60]. Current research is focusing on whether entrectinib is also suitable for pediatric CNS tumors [188].

### NTRK fusions

Members of the NTRK family of RTKs are especially highly expressed in neural tissue [6, 185]. These receptors participate in the development and proper functioning of the CNS. The NTRK family exists of three members, NTRK1, NTRK2, and NTRK3, which besides the RAS/MAPK pathway can also signal via the PI3K/AKT/mTOR and the PLCγ/PLK pathways, depending on which docking protein binds to the kinase domain. Via these pathways, the signal transduction leads to proliferation, prevention of neuron degeneration, development, synaptic plasticity, sensory neuron maintenance and neuronal differentiation [108, 131, 156, 211]. These receptors and their signaling cascade are also implicated in neoplastic cells [156].

Whether NTRK fusions signal via the same preferred pathways as their full-length counterparts is still unknown. Experiments with the ETV6–NTRK3 fusion showed that this fusion protein signals mainly through RAS/MAPK but also activates PI3K/AKT/mTOR. Activating both pathways might induce the oncogenic potential since it activates proliferation and inhibits apoptosis [152, 223].

Although mutations and alternative splicing occur, fusions are the most common aberrations of NTRK in tumors. The most common alteration is a fusion between an *NTRK* gene and another N-terminal partner [71, 143, 219, 224, 234]. All these aberrations result in the constitutive activation of the kinase, due to loss of the extracellular domain. Of the 80 different N-terminal partners observed in tumors [80], 22 occur in pediatric CNS neoplasms (Online Resource 6). NTRK fusions have been identified in several pediatric gliomas such as pilocytic astrocytoma, high-grade glioma and glioblastoma (Fig. 3b) [19, 40, 93, 97, 116, 135, 177, 182, 184, 192, 240, 245]. In contrast to other tumor types, NTRK2 is the most common fusion partner of the NTRK family in pediatric brain tumors [215, 229, 234]. NTRK fusions belong to the type I kinase fusions as in 16/22 (73%) NTRK fusions the N-terminal partner has at least one coiled-coil or other type of dimerization domain (Fig. 2b). This probably leads to a constitutively active kinase, continuous downstream signaling and thus proliferation and cell survival. However, N-terminal partners such as CHTOP and VCL do not have a dimerization domain or another oncogenic functional domain. For these fusions it might be possible that the loss of the regulatory domain in the N-terminus of NTRK is enough to drive oncogenesis or there might be another yet undiscovered mechanism.

While the percentage of NTRK fusion driven tumors is quite low, the incidence in pediatric HGG and diffuse infiltrating pontine glioma is around 5% and even 40% in infants with non-brainstem HGG [163, 240]. NTRK inhibitors could be a potential effective targeted therapy in these tumors. Furthermore, these NTRK inhibitors have already shown high efficacy in several case reports of NTRK fusion driven tumors [49, 52, 57, 120, 153, 195, 209, 252]. Recent phase I and II trials have confirmed the effectivity of larotrectinib and entrectinib in pediatric brain tumors. Moreover, larotrectinib is now approved for NTRK fusion positive tumors in pediatric patients [45, 51, 117, 122, 173].

### FGFR fusions

The FGFR family exists of four transmembrane tyrosine kinase receptors (FGFR1–4). The FGFR family plays an important role in embryonal CNS development as well as in tumorigenesis, regulating angiogenesis, proliferation, differentiation, migration, and survival. Aberrant signaling of the FGFR family is seen in many different cancers as a result of SNVs, overexpression or rearrangements (reviewed in [230]). FGFR fusions have been identified as oncogenic drivers in brain, bladder, lung and breast tumors. In pediatric CNS tumors nine different FGFR fusions have been identified (Online Resource 7).

All FGFR fusion proteins retain the kinase domain of FGFR and almost all C-terminal partners contain a coiled-coil domain. This means FGFR fusions belong to type III kinase fusions (Fig. 2b). Other oncogenic mechanisms are also proposed, for example, the FGFR3–TACC3 fusion displaces FGFR3 to the mitotic spindle leading to aneuploidy and tumorigenesis [178, 210]. Simultaneously, the constitutively active signal of FGFR3 also transduces via the RAS/MAPK pathway [158]. In contrast to other RTK fusion proteins, FGFR fusions depend on their own promoter for the expression of the chimeric protein. FGFR3 is very lowly expressed in normal brain and fusion negative adult glioblastoma but is highly expressed in fusion positive glioblastoma, which is likely due to the loss of microRNA regulation [174, 199]. The 3′ UTR of the *FGFR3* gene is negatively controlled by microRNAs in the normal brain. However, in the fusion gene, this region is lost and *FGFR3* can thus no longer be controlled by *mir-99a* [174]. The sequence of the 3′ UTR regions in *FGFR* genes is quite diverse but is lost in all the fusion genes. Additionally, computational analysis has shown that these UTRs are presumably regulated by different miRNAs. Therefore, it is likely that the loss of the 3′ UTR of *FGFR* in fusion genes leads to the enhanced expression of the chimera [94]. To date, no fusions have been detected with family member FGFR4 in pediatric CNS neoplasms. Furthermore, fusions with FGFR1 and FGFR3 often occur due to a small deletion on the chromosome, fusing them to partners in close proximity. In contrast, FGFR2 often translocates to partners on other chromosomes [175].

Since new aberrations in the FGFR pathway are detected in a variety of tumor types, there is an interest in FGFR pathway inhibitors. Clinical trials with FGFR inhibitors in brain tumors are being conducted [48, 92]. The FGFR inhibitor ponatinib has an improved therapeutic activity of temozolide in in vitro patient derived DIPG cells [200]. Further research will show the efficacy of the FGFR inhibitors in the different FGFR fusion positive pediatric tumors.

### MET fusions

The least common RTK fusions in pediatric CNS neoplasms are fusions involving MET. In the three different MET fusions in pediatric CNS tumors, CLIP2-MET, TFG-MET, and PTPRZ1-MET, the kinase domain of MET is retained within the fusion (Online Resource 8). In CLIP2-MET and TFG-MET, only the C-terminal part of the protein containing the kinase domain is retained and therefore lacks its autoregulatory domain leading to a constitutive active MET [19, 141]. MET fusions can thus be classified as type II kinase fusions (Fig. 2b). Additionally, TFG has also been described as a partner for RTKs in chimeric proteins in other neoplasms [72, 77]. The PTPRZ1–MET fusion has been described in adult glioblastomas and entails the full length MET protein fused to the first exons of PTPRZ1 and probably uses its promoter to overexpress MET [17]. Additional to the MET fusions, all patients also harbored *TP53* mutations or *CDKN2A* and *CDKN2B* deletions, indicating that the tumorigenesis due to MET fusions is probably dependent on additional aberrations in the cell cycle regulation [19]. It is shown that in a preclinical setting MET inhibition with RTK inhibitors is effective and MET fusion positive pediatric glioblastoma respond positively before relapse [19]. This initial data promotes further research with MET inhibitors in pediatric glioblastomas.

## Fusions involving transcription regulators in pediatric CNS tumors

**Fig. 4:**
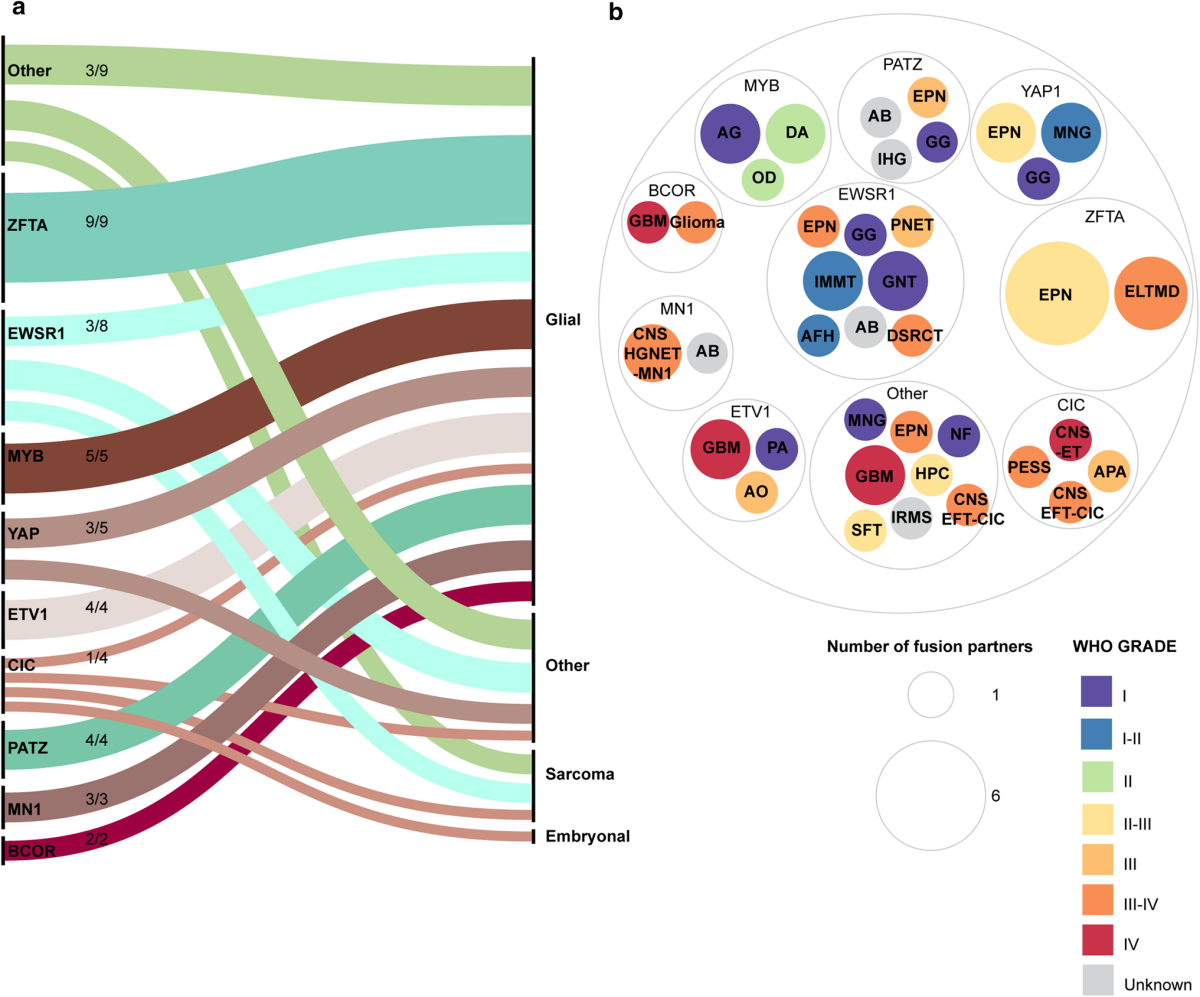
Characteristics of transcription regulator fusions in pediatric CNS neoplasms **a** Nine transcription factors are responsible for most transcription factor fusions that are mainly present in glial tumors. **b** Kinase proteins are not limited to one pediatric CNS tumor type but are present in multiple different tumor types. Graph made by https://app.rawgraphs.io. *AB* astroblastoma, *AFH* angiomatoid fibrous histiocytoma, *AG* angiocentric glioma, *APA* anaplastic pleomorphic astrocytoma, *AO* anaplastic oligodendroglioma, *CNS EFT–CIC* central nervous system Ewing Sarcoma Family Tumor with CIC alteration, *CNS—ET* central nervous system embryonal tumor, *CNS-HGNET MN1* central nervous system high grade neuroepithelial tumor with MN1 alteration, *DA* diffuse astrocytoma, *DSRCT* desmoplastic small round cell tumor, *ELTMD* ependymoma like tumor with mesenchymal differentiation, *EPN* ependymoma, *GBM* glioblastoma, *GG* ganglioglioma, *GNT* glial neuronal tumor, *HPC* hemangiopericytoma, *IHG* interhemiscpheric glioma, *IMMT* intracranial myxoid mesenchymal tumor, *IRMS* intracranial rhabdomyosarcoma, *MNG* meningioma, *NF* neurofibroma, *OD* oligodendroglioma, *PA* pilocytic astrocytoma, *PESS* primary epidural spinal sarcoma, *PNET* primitive neuroectodermal tumor, *SFT* solitary fibrous tumor

There are 38 fusions reported in 53 fusion–entity pairs, that do not have a kinase domain. Most of these (94%; 50/53), however contain a transcription activation domain, indicating that transcription regulators also play an important role in oncogenic fusions in pediatric CNS tumors. There are three (6%) fusion–entity pairs with two fusions that do not belong to this group and only have uncharacterized domains: RNF213–SLC26A11, and SETD2–ROBO1. Nine fusion partners are responsible for the majority of fusions (86%; 43/50) driven by transcriptional regulators (Fig. 4a). These nine transcription regulators occur with multiple partners, while the remaining transcription regulators have only one fusion partner and account for the remaining seven fusion–entity pairs: NAB2–STAT6, ATXN–NUTM1, PAX3–NCOA1 and PLAGL1–FOXG1, these will not be discussed further (Online resource 1). Transcription regulator fusions mostly occur in the glial tumor types (69%; 37/53) (Fig. 4a). The different fusions are not exclusively present in certain tumor types (Fig. 4b).

Overall, we have identified two different types of transcription regulator fusions. Most of these fusions contain at least one partner that functions as a transcription factor and retains its transcription activation domain, while the other partner retains its nuclear localization signal (NLS) as well as, sometimes, a DNA binding domain (Fig. 2b, IV). An example of this kind of fusion is ZFTA–RELA (Fig. 2c, IV). In fusion type V, the transcription activation and DNA binding domain as well as the NLS are retained within one fusion partner, while the other partner influences the regulation of the chimeric protein. This is also seen in MYB–PCDHGA1, where PCDHGA1 is responsible for the truncation of the negative regulation domain in MYB (Fig. 2b V, c V). For BCOR fusions the mechanism of the chimeric proteins is unknown.

Furthermore, many of these fusions make use of epigenetic mechanisms to activate transcription of the alternative target genes to induce tumorigenesis. Hence, more research in epigenetic mechanisms could aid the overall understanding of these tumors and the identification of potential therapeutic treatments. As these fusions partners themselves are often difficult to target, it is worth identifying targetable downstream factors.

### ETV1 fusions

ETS variant transcription factor 1 (ETV1) belongs to the ETS family of transcription factors. This family regulates genes that are responsible for processes such as cell growth, angiogenesis, proliferation and differentiation [102]. Furthermore, these transcription factors are known oncogenes in Ewing sarcoma, melanoma and prostate cancer [12, 88, 89]. It is unclear what the exact oncogenic mechanism is of DGKB–ETV1 fusions in glioblastoma and PTPRZ1–ETV1 in glioblastoma, pilocytic astrocytoma and anaplastic oligodendroglioma (Fig. 4b, Online Resource 9). However, as elevated expression levels of ETV1 are detected in multiple tumor types and can lead to increased invasiveness of tumor cells, this might also be the oncogenic mechanisms of these fusions [93, 146]. The promoters of *PTPRZ1* and *DGKB* are highly active in the CNS and the DNA binding domain of ETV1 is retained in the fusion, possibly leading to an enhanced activation of ETV1 target genes [19]. As ETV1 itself retains its NLS, DNA binding domain and its transactivation domain, it belongs to the type V transcriptional regulator fusions.

### EWSR1 fusions

EWSR1 is an RNA binding protein that plays an important role in transcription initiation. The protein is vital for cell survival in the CNS and regulates genomic integrity and RNA maturation processes [146]. Translocations with different partners are seen in several cancers, mainly soft tissue sarcomas. EWSR1 most often fuses with transcription factors. The new chimeric proteins alter pathways that are important for cell growth, differentiation, and proliferation, which ultimately leads to tumorigenesis [147]. In pediatric CNS tumors five different EWSR1 fusions have been detected, such as EWSR1–PLAGL1 and EWSR1–CREB1 (Online Resource 10). In all these fusions the transcription activation domain of EWSR1 is retained as well as the DNA binding domain (zinc finger, leucine zipper) and the NLS of its partners, making the fusions part of the type IV transcription regulator fusions. Additionally, to these type IV fusions, there is one EWSR1–SMARCA5 fusion in a Ewing sarcoma/primitive neuroectodermal tumor [205, 217]. Apart from its DNA binding domain (SANT) and its NLS, SMARCA5 also retains its helicase domain, which might implicate an epigenetic function in tumorigenesis for this fusion [24]. At the moment due to the difficulties in targeting transcription regulators, there are no targeted therapies available for EWSR1 fusions [187].

### ZFTA fusions

ZFTA–RELA is the most recurrent fusion in ST-EPN. Around 70% of the ST-EPNs are driven by this fusion and EPNs with this fusion belong to the subtype ST-EPN–ZFTA. While ZFTA is a poorly characterized transcription factor, RELA (p65) is important in the canonical NFκB pathway. Initially, oncogenicity of the fusion was attributed to NFκB activation by RELA. However, due to identification of five additional fusion partners for ZFTA (MAML2–3, NCOA1–2 and YAP1), the poorly characterized ZFTA seems to be the driving fusion partner (Online Resource 11) [105, 115, 176, 226, 249, 255]. All these C-terminal partners are transcription factors or co-activators like RELA. This leads to the hypothesis that the zinc finger domains as well as the NLS in ZFTA are important for the oncogenic action of the fusion, as is seen in type IV transcription regulator fusions. This zinc finger domain and its interactions might alter the trafficking, degradation or target specificity of the chimeric fusion partner and thereby altering the transcription of their targets [176, 251]. Recent research has shown that the zinc finger domain in ZFTA is indeed essential for tumorigenesis [249]. More specifically, the zinc finger is responsible for nuclear translocation of the fusion protein, the binding to chromatin and the recruitment of chromatin remodeling complexes. This way ZFTA binds its fusion partners across the whole genome, modifies the chromatin state and facilitates transcriptional co-activators to promote expression of oncogenic genes [9, 115]. All ZFTA fusion partners retain their transcription activation domain, thus being able to enhance the activation of these oncogenic genes. In utero electroporations with ZFTA fusions in mice have confirmed that induction of the fusion alone is sufficient to drive ependymomas in vivo [249]. These studies also show that drugs can potentially be directed to genes in the downstream signaling cascade of ZFTA-fusions. Further research is needed to see if ZFTA positive tumors can be therapeutically targeted in this way. Additionally, therapeutics should be investigated that can cause a fast degradation of ZFTA (-fusions) [9, 115]. Recently, ZFTA fusions have also been discovered in tumors that although morphologically and genetically resemble ependymomas, do not cluster together with ST-EPN based on their DNA methylation profile [226]. Hence, for now they are represented as a separate type called ependymoma-like tumors with mesenchymal differentiation (ELTMDs). This data, together with the expression of ZFTA fusions in chondroid lipomas shows that ZFTA fusions might be oncogenic drivers in multiple diseases, also outside of the CNS [61, 226].

### YAP1 fusions

YAP1 fusions are also mostly detected in ST-EPN, although less frequently than ZFTA–RELA. YAP1 is a regulator of the Hippo pathway and can fuse to mastermind-like proteins MAMLD1 and MAML2 or a thus far uncharacterized protein called FAM118B (Online Resource 12). No other recurrent aberrations have been identified in addition to these fusions, making the fusion the likely oncogenic driver. Indeed, in vivo experiments have demonstrated that ectopic expression of the YAP1–MAMLD1 fusion in fetal mouse brain is sufficient to induce tumors [168]. YAP1 and the Hippo pathway are responsible for the limitation of organ growth and can promote tumorigenesis. Under normal conditions YAP1 is retained in the cytosol until it is activated by the Hippo pathway and translocated to the nucleus, where YAP1 with its TEAD domain acts as a transcriptional activator. In fusions, YAP1 is retained in the nucleus and therefore has an oncogenic potential [151, 247]. The NLS in MAMLD1 is necessary for the retention of YAP1 and its transactivation domain in the nucleus. YAP1–FAM118B is also retained in the nucleus, although the exact mechanism here is unclear since the NLS of FAM118B fused to YAP1 is not enough to drive ependymoma [168]. MAMLD1 thus might have an additional function that drives oncogenesis apart from the nuclear localization. A YAP1 fusion with MAML2 has been detected in meningiomas. Where the TEAD domain of YAP1 is also retained as well as the transcriptional activation domain of MAML2, leading to the co-activation of the Hippo pathway [206, 241]. As most of the YAP1 fusions make use of the NLS of the fusion partner, these fusions are part of the type IV transcription regulator fusions.

### CIC fusions

Capicua transcriptional repressor (CIC) is a transcription factor and in pediatric brain tumors a fusion was initially reported between CIC and NUTM1 in CNS Ewing sarcoma family of tumors with *CIC* alterations (CNS EFT–CIC) [216]. Additionally, fusions between CIC and LEUTX have been detected in an anaplastic pleomorphic astrocytoma and a CNS embryonal tumor (Fig. 4b, Online Resource 13). Recently, a CIC fusion with DUX4 was detected in a single case of primary epidural spinal sarcoma [50]. Although, this is a known fusion in Ewing sarcomas, it has never been detected in the CNS and in contrast to the CNS EFT–CIC tumors, the tumor was located in the spinal cord and not in the cerebrum. It remains to be investigated whether all CIC fusions belong to the same CNS entity or whether they may represent distinct entities or specific subtypes.

As also seen in CIC–NUTM1 and CIC–LEUTX fusion proteins, the chimeric CIC–DUX4 protein retains most of the functional regions of CIC, including the DNA-binding high-mobility group (HMG) box and the MAPK phosphorylation sites, while the partners retain their TEAD domain [50, 216]. This is seen in type IV transcription regulator fusions and might indicate that these domains are important for the oncogenic potential of the fusion. CIC is a transcriptional repressor that prevents activation of genes downstream of RTK signaling. In oligodendrogliomas, mutations in this gene are correlated with a poor outcome. These mutations lead to a loss-of-function and thus the activation of downstream RTK signaling. It is hypothesized that in the CIC fusions the downstream signaling is also activated by the recruitment of chromatin modifiers such as histone acetyl transferases to the transcription activation domains of the fusion partners [65, 216].

### MN1 fusions

MN1 proto-oncogene, transcriptional regulator (MN1) is a transcriptional coregulator that has rearrangements in meningioma and leukemia [78, 125]. The first MN1 fusions located in the CNS were described in an entity named central nervous system high-grade neuroepithelial tumor with *MN1* alterations (CNS HGNET–MN1) [216]. Recently, 73 CNS HGNET–MN1 tumors have been analyzed [32]. Most of these tumors have an oncogenic fusion between MN1 and BEND2 (Online Resource 14). However, the partner CXXC5 is also commonly present and additionally an MN1–GTSE1 fusion has been detected. In the MN1–BEND2 fusion, the transactivating domain of MN1 is retained as well as the BEN domains of BEND2. These BEN domains are thought to play a role in DNA binding, chromatin organization and neural transcriptional regulation [43, 196, 216]. The transactivation domain of MN1 is thought to recruit transcription activators, although, the exact oncogenic mechanism is unknown [186]. For MN1–CXXC5 and MN1–GTSE1 no research has been done into the chimeric fusion structure and the oncogenic mechanism. It is known that CXXC5 retains its NLS and DNA binding domain as also seen in BEND2, and GTSE1 retains its NLS, making MN1 fusions part of the type IV transcription regulator fusions. Recently, in the new 5th edition of WHO classification of CNS tumors, the HGNET–MN1 tumors have been renamed into astroblastoma, *MN1* altered, as most of these tumors show an astroblastoma morphology [124, 137, 138]. It remains to be investigated whether tumors with MN1–BEND2 or MN1–CXXC5 fusions indeed all belong to this same entity or to what extent they may differ from each other molecularly and/or clinically.

### PATZ1 fusions

PATZ1 (POZ/BTB and AT hook containing zinc finger 1) is a transcription factor and is important in maintaining pluripotency and hindering differentiation in stem cells [166]. Additionally, at different expression levels of PATZ1, the protein can act as either a transcriptional repressor or activator influencing senescence and proliferation, respectively [140]. Two different PATZ1 partners, EWSR1 and MN1 have been identified in several different pediatric CNS neoplasms (Online Resource 15). As described above, both EWSR1 and MN1 are implicated in other tumor entities as well and positively influence transcription regulation [10, 125]. Although the MN1–PATZ1 tumors also contain MN1 fusions, DNA methylation data of these tumors do not cluster together with the other above mentioned astroblastoma, *MN1* altered tumors [22]. While the exact oncogenic mechanism of the PATZ1 fusion proteins is still unknown, it is known that the transactivation domain of MN1/EWSR1 is retained as well as the zinc finger domain of PATZ1. PATZ1 fusions thus belong to type IV transcription regulator fusions. It is hypothesized that this leads to enhanced activation of genes near the DNA binding site from PATZ1, via the recruitment of the transcription activators to the transactivation domain. Additionally, PATZ1 might benefit from the elevated expression levels by using the promoter of EWSR1 and MN1 [5]. However, a PATZ1–MN1 fusion was also identified, indicating that the aberrant expression is not the only oncogenic mechanism [255].

### BCOR fusions

BCL6 corepressor (BCOR) epigenetically silences different genomic regions and is important in embryonal development. Several aberrations in BCOR have been implicated in different tumor types, including internal tandem duplications in CNS high-grade neuroepithelial tumors with *BCOR* alterations (CNS HGNET–BCOR) [216]. In the last years, two different fusions with BCOR have been identified in pediatric brain tumors (Online Resource 16). Interestingly, the two fusion partners are paralogues of each other. EP300–BCOR and BCOR–CREBBP have been described as potential oncogenic drivers in gliomas [181, 228]. EP300 and CREBBP are both histone acetyltransferases, which are important in proliferation, differentiation and may even have tumor suppressor functions. For EP300–BCOR several different fusion sites have been identified. In two of the three known fusions, the transactivation domain is truncated, and the acetyltransferase domain is retained, in the third fusion all domains are retained [228]. In the BCOR–CREBBP fusion the acetyltransferase domain is lost, and it probably creates a premature stop codon in CREBBP. The reciprocal *CREBPP*–*BCOR* fusion was not detected [181]. Since the order of the partners within the chimeric protein is different and the retention of the domains varies between the different fusions, it is difficult to predict the fusions’ oncogenic mechanism.

### MYB fusions

MYB alterations and fusions have been identified in p-LGG for the first time in 2010 [222]. This transcription factor plays a role in the proliferation and differentiation of hematopoietic and other progenitor cells. An oncogenic effect has already been described in both leukemia as well as solid tumors. Ten percent of p-LGG harbor MYB alterations, with the most common alteration being a MYB–QKI fusion [15]. DNA methylation data of pediatric gliomas with a MYB or MYBL alteration cluster together as one entity [33], which is now classified as a new subtype of diffuse gliomas: diffuse astrocytoma—MYB altered [54, 137, 138]. The mechanism behind the MYB–QKI fusion has been well studied and is proposed to be a tripartite mechanism: MYB is overexpressed, the regulatory domain of MYB is truncated and the tumor repressor function of QKI is lost. There are two different fusion sites for the MYB protein in MYB–QKI, one in which the regulatory domain is truncated and one in which it is retained. Other fusion partners of MYB in pediatric gliomas have also been identified: ESR1, MAML2, MMP16 and PCDHGA1 (Online Resource 17). However, not much is known about these fusions. The hypothesis is that these fusions have an oncogenic potential due to the truncation of the C-terminal end of the MYB protein, meaning they belong to type V transcription regulator fusions, but there might also be other mechanisms involved. As seen in MYB–ESR1, where the negative regulation domain of MYB is retained as well as the ESR1 co-activator domain. Currently, there are no small molecular inhibitors for MYB and their development is rather challenging. However, transcriptional targets of MYB fusion proteins could be targeted [15]. Further research is necessary to determine whether similar MYB target genes are activated by the different chimeric proteins.

## Validation of fusion proteins as oncogenic drivers

Over the coming years, a variety of fusions will likely be detected by NGS, as this detection mode is becoming standard care in diagnostics. Additionally, pan-cancer studies combining sequencing data from all over the world, can identify many new fusion genes in different tumor types. Moreover, for the fusions that have already been detected, there is still a lack of experimental evidence for the oncogenic potential*. *In vitro and in vivo*,* only a few of these fusions have been validated, some of which only in models that are not pediatric brain tumors but do give an indication of the oncogenic potential (Table 1). Only 30 of the 110 fusion proteins presented in this review have been validated in an in vitro or in vivo model.

**Table 1:** In vitro and/or in vivo validated oncogenic fusion proteins that drive pediatric CNS neoplasms

Fusion	In vitro model	References	In vivo model	References
FAM131B–BRAF	Transforms NIH3T3	[39]		
KIAA1459–BRAF	Constitutive kinase activation in Cos-7 cells and transforms NIH3T3	[98]	Transduced ﻿Wild-type and Tsc1 − / − NSCs injected into C57/Bl6 mice cerebellum	[104]
NFIA–RAF1	MAPK activation in HEK293 cells, transforms HeLa	[242]		
QKI–RAF1			QKI–RAF1 expressing PMAs intracranially injected into cerebral cortex of NOD scid gamma mice	[86]
			NIH3T3-expressing QKI–RAF1 injected into flanks of NSG mice	[86]
SRGAP3–RAF1			SRGAP3–RAF1 expressing PMAs intracranially injected into cerebral cortex of NOD scid gamma mice	[86]
			NIH3T3-expressing SRGAP3–RAF1 injected into flanks of NSG mice	[86]
CCDC88A–ALK			Transduced astrocytes orthotopically injected in brains of NOD/SCID/NSG mice	[74]
PPP1CB–ALK			Transduced astrocytes orthotopically injected in brains of NOD/SCID/NSG mice	[74]
			CD1 mice in utero electroporated	[40]
KIF5B–ALK	Transforms NIH3T3	[35]		
EML4–ALK	Transforms NIH3T3	[35]	Transgenic mice for NSCLC	[213]
			Transgenic mice for NSCLC	[183]
CEP95L–ROS1	Transforms NIH3T3, immortalized astrocytes and Ba/F3	[44]		
GOPC–ROS			Conditional GOPC–ROS transgenic mice	[31]
KLC1–ROS1	Transforms NIH3T3 and GBM cells	[157]		
TPM3–NTRK1			Transduced TP53-null astrocytes transplanted into brain of immunodeficient mice	[240]
BTBD1–NRTK3			Transduced TP53-null astrocytes transplanted into brain of immunodeficient mice	[240]
ETV6–NTRK3	Transforms NIH3T3	[237]	Transduced NIH3T3 subcutaneously injected in SCID mice	[237]
	Transforms Ba/F3	[139]	Transduced HMLER cells transplanted in athymic mice	[139]
FGFR1–TACC1	Transforms Rat1A cells	[210]	Transduced Ink4A;Arf − / − astrocytes subcutaneously injected in immunodeficient mice	[210]
FGFR2–INA	Transforms NIH3T3 and PMA	[87]		
FGFR3–TACC3	Transforms Rat1A cells	[210]	Transduced Ink4A;Arf − / − astrocytes subcutaneously injected in immunodeficient mice	[210]
			Glioma cells intracranially xenografted in immunodeficient mice	[174]
CLIP2–MET			GBM cells with fusion transplanted in SCID mice	[19]
PTPRZ1–MET	MAPK activation in HEK293T cells	[19]		
	Transforms GBM cells	[17]		
TGF–MET			Ntv-a; Cdkn2a − / − ; Pten fl/fl animals injected with RCAS–TFG–MET	[19]
EWSR1–SMARC5A	Transforms NIH3T3	[217]		
ZFTA–MAML2			CD1 mice in utero electroporated with plasmid mix containing pT2K–IRES–luc–ZFTA–MAML2 and a pCAGGS plasmid with the Tol2 transposase	[249]
ZFTA–MAML3			CD1 mice in utero electroporated with plasmid mix containing pT2K–IRES–luc–ZFTA–MAML3 and a pCAGGS plasmid with the Tol2 transposase	[249]
ZFTA–RELA			NSC from transgenic mice transplanted into CD1-nude mice	[175]
			In utero electroporation of ﻿pBCAG–HA–ZRFUS1, pbCAG–eGFP, pX330–438 sgTp53, GLAST–PBase, pBCAG–Luc in the lateral ventricles	[9]
			Transduced mouse NSCs ortotopically allografted	[115]
			CD1 mice in utero electroporated with plasmid mix containing pT2K–IRES–luc–ZFTA–RELA and a pCAGGS plasmid with the Tol2 transposase	[249]
			GFAP (G)/tv-a, Nestin (N)/tv-a (agouti), N/tv-a;Ink4a-Arf-/-;Pten fl/fl,or BLBP (B)/tv-a mice intracranially injected with RCAS ZFTA–RELA plasmid	[67]
YAP–FAM118B			GFAP (G)/tv-a Cdkn2a wild type, G/tv-a;Cdkn2a-null, or Nestin (N)/tv-a;Cdkn2a-null mice intracranially injected with RCAS YAP–FAM118B plasmid	[218]
YAP1–MAMLD1			CD1 mice in utero electroporated	[168]
			CD1 mice in utero electroporated with plasmid mix containing pT2K–IRES–luc–YAP1–MAMLD1 and a pCAGGS plasmid with the Tol2 transposase	[249]
			GFAP (G)/tv-a Cdkn2a wild type, G/tv-a;Cdkn2a-null, or Nestin (N)/tv-a;Cdkn2a-null mice intracranially injected with RCAS YAP–MAMLD1 plasmid	[218]
MYB–QKI			Transduced NIH3T3 subcutaneously injected in NSG mice	[15]
			Transduced NIH3t3 intracranially injected in ﻿immunocompromised ICR–SCID mice	[15]

## Fusion genes in diagnostics

With the recent 5th edition of the WHO classification for tumors of the CNS, in which they advance the role of molecular diagnostics for CNS tumors, the detection of fusion genes has become an important diagnostic marker in pediatric CNS neoplasms [137]. Routinely, targeted assays were being used for diagnostic purposes to detect these fusions. Although these assays are cost-effective, they have clear limitations. As only specific fusions and breakpoints are included within these assays, alternative breakpoints, additional fusions or alternative fusion partners are likely to remain undetected. Additionally, targeted assays are unable to discover completely novel oncogenic fusions [193]. More recently, RNA-sequencing (RNA-seq) is being implemented in the diagnostic setting to detect oncogenic gene fusions, using both fresh frozen and formalin fixed paraffin embedded material [18, 119]. RNA-seq is a robust way to pick up expressed fusion RNAs, but calling oncogenic fusions remains challenging. First, there are currently no standardized methods with multiple different algorithms being used.

Second, next to true oncogenic fusions, fusion calling algorithms pick up a lot of false positives such as read-throughs and non-malignant fusions. Determining the cut off in fusion calling algorithms is a manual task that can introduce a bias. The difficulty here is to detect all the real fusion drivers, while ignoring biological and algorithm artefacts [26, 148]. Fusion panels can make detection of fusions less complex in RNA-seq by limiting the analysis to key genes associated with aberrations in pediatric CNS neoplasms. These panels can aid the molecular characterization of the tumors as well as contribute to the therapeutic decision making [119]. While these panels can detect fusions with different breakpoints or partner genes and could thus be used for diagnostics, they are limited to the number of genes in the panel and will thus neglect completely novel fusions with two yet unknown partners. An alternative to distinguish real fusions from artefacts is inclusion of genomic data, such as whole genome or exome sequencing to pinpoint underlying structural variations. This multi-omics approach can aid the assessment of the potential pathogenicity of the fusion and the clinical decision making, especially for lowly expressed oncogenic fusions [18].

## Discussion

Fusion proteins as oncogenic drivers are emerging in pediatric CNS neoplasms. Since the implication of NGS more of these drivers have been described in case studies as well as in big multi-center sequencing studies. However, the variety of these fusions, the range of tumor entities in which they can occur, and their molecular mechanism are for a large part still unknown. Literature reveals that most fusions have an active kinase domain, and these kinase fusions are driven by a few main partner genes that are responsible for the oncogenicity of the fusion. We observed that there is a growth in detection of the variety of partners for these main oncogenic partners and we expect that in the next years this variety will only further increase. In line with this, the amount and diversity of non-kinase fusion proteins has also advanced. Transcription regulators now comprise the second biggest group of fusion proteins. These fusions are mainly seen in tumor entities that have been recently discovered or reclassified. With the discovery of these transcriptional regulator fusions probably more brain tumor entities can be reclassified based on their molecular mechanism. Although, this review did not identify the prevalence of these different fusions, the reclassification of the tumor entities based on the fusion that is present, as seen in many of the transcription regulator fusions, shows that the fusions play an important part in these entities. Future research should focus on the mechanism behind these fusions to identify targeted therapeutic options for the distinct tumor entities.

## Supplementary Information

Below is the link to the electronic supplementary material.

Supplementary file1 (XLSX 24 kb)

Supplementary file2 (PDF 556 kb)
