# Supplementary material for: The oncogenic fusion landscape in pediatric CNS neoplasms
Source: Acta Neuropathol. 2022 Feb 15;143(4):427–51. doi: 10.1007/s00401-022-02405-8 (PMC8960661; doi:10.1007/s00401-022-02405-8)
Supplement: Supplementary file 2 — Supplementary file2 (PDF 556 kb) [file 401_2022_2405_MOESM2_ESM.pdf]

Online resource 2

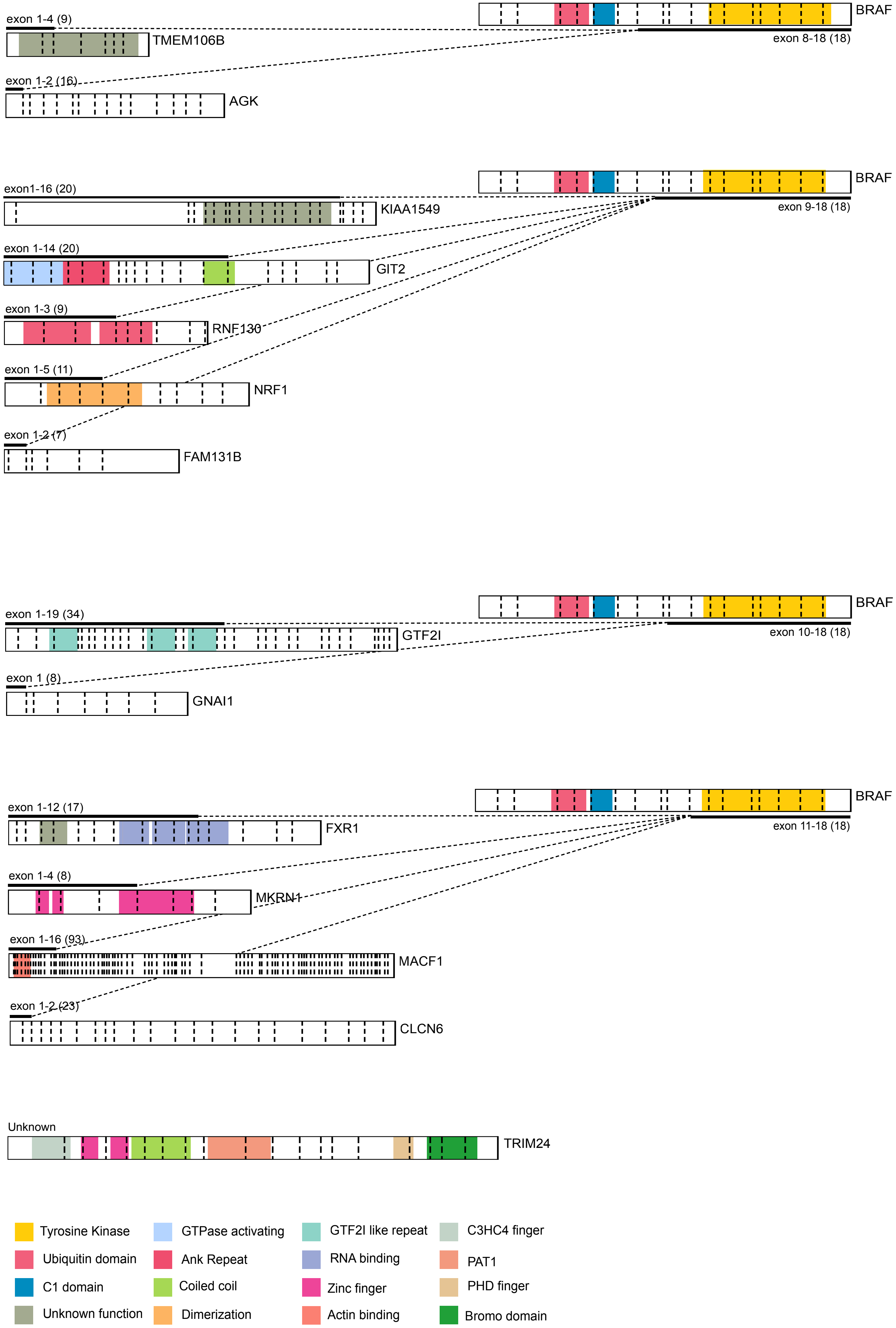

Online resource 3

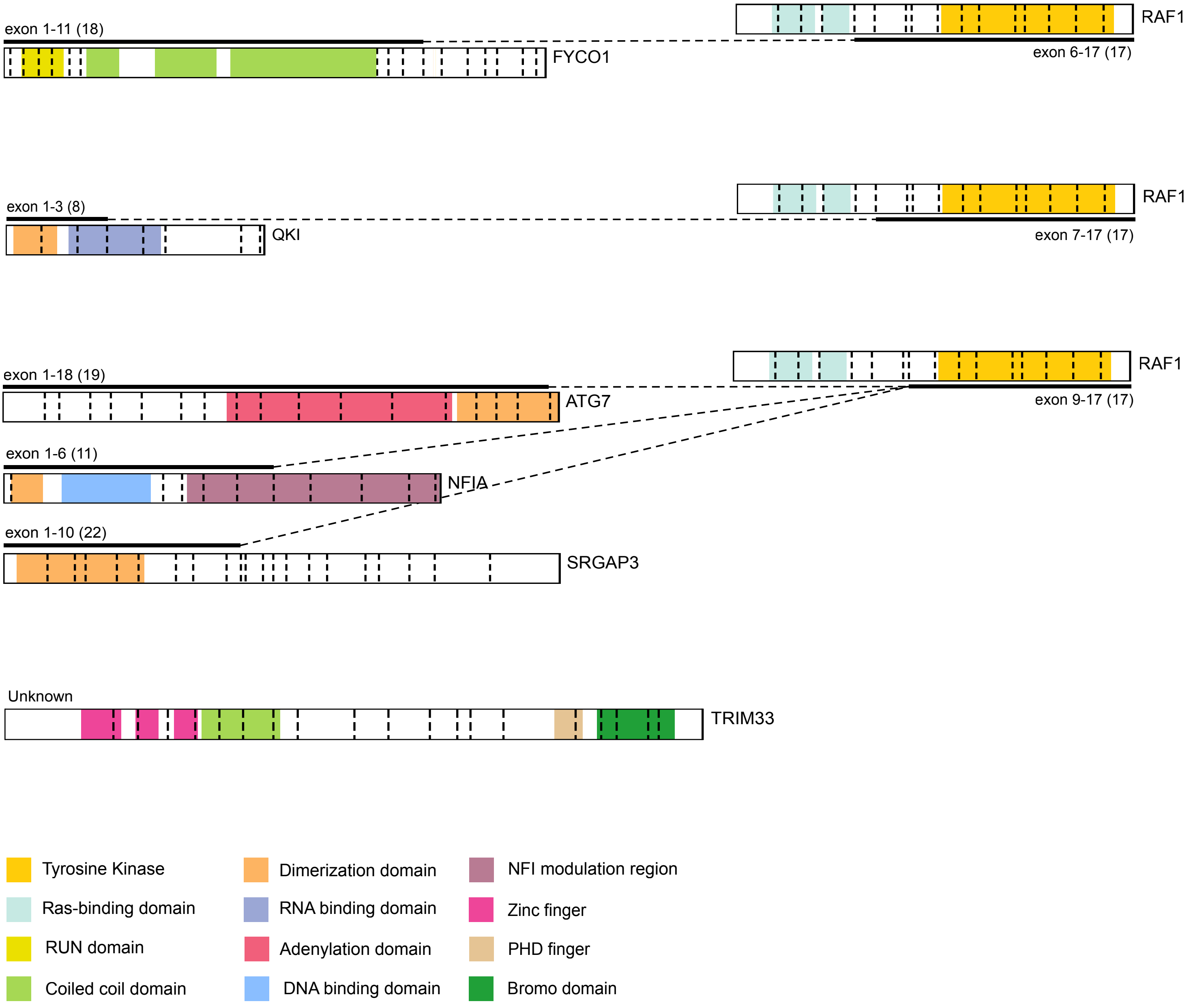

Online resource 4

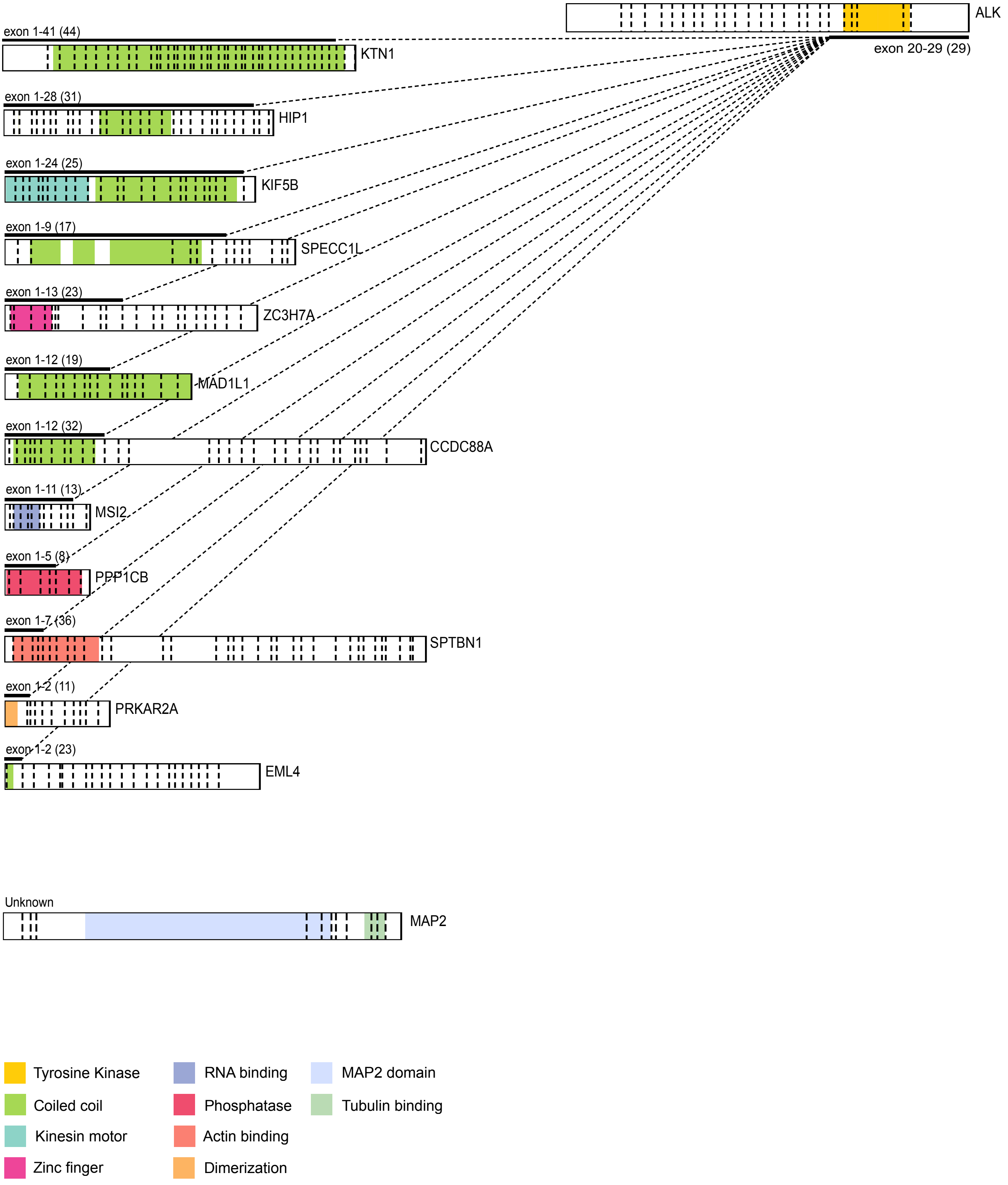

Online resource 5

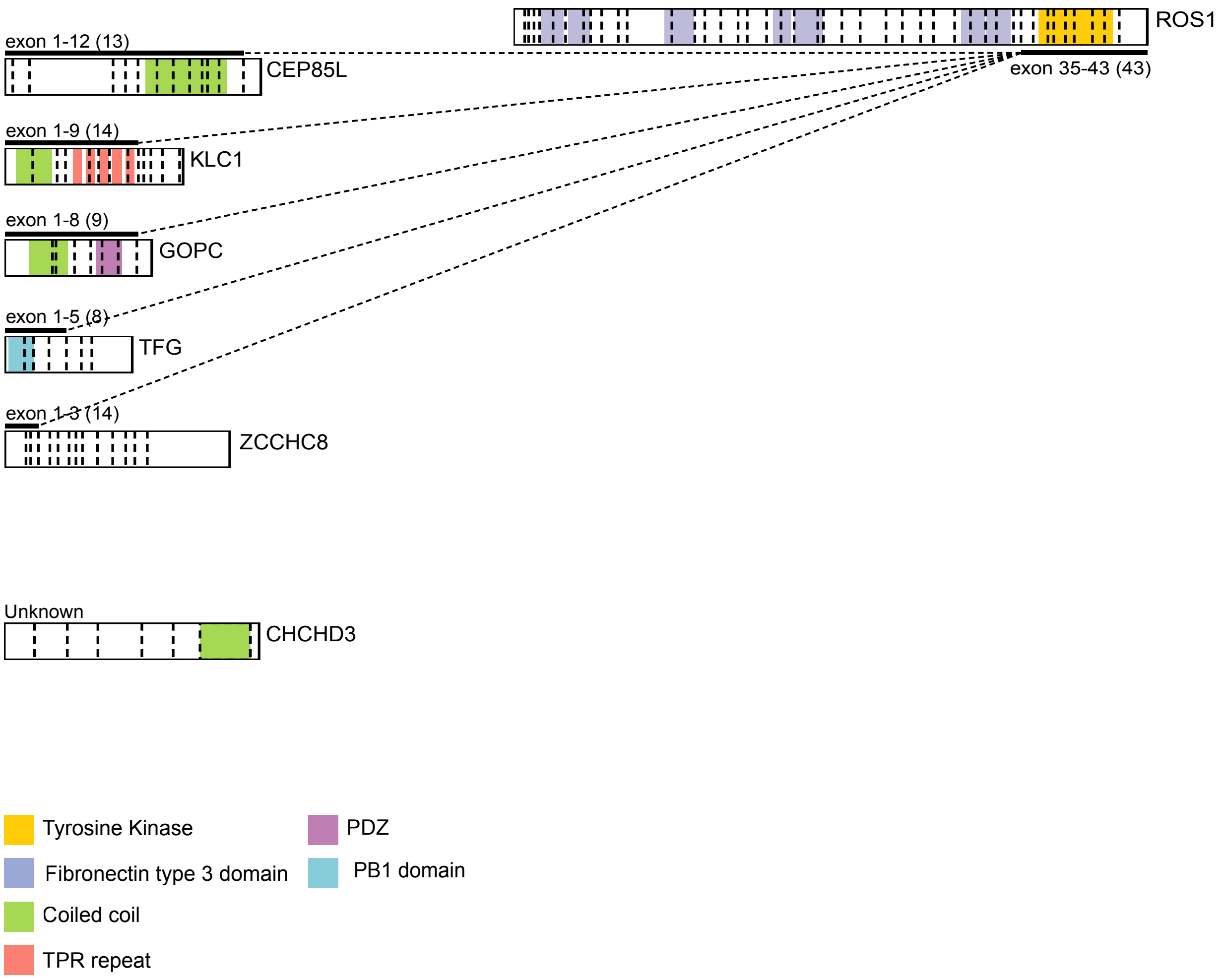

Online resource 6a

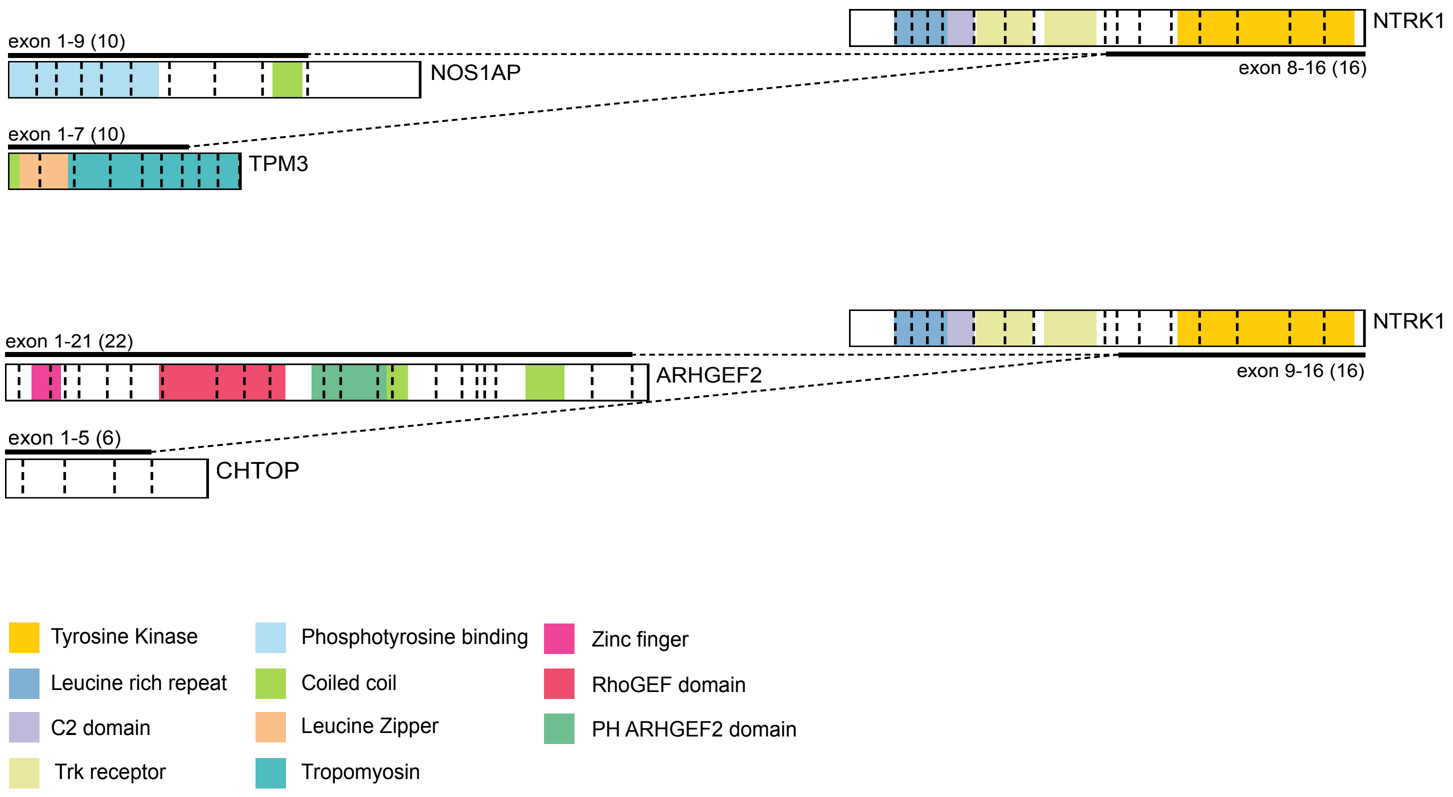

Online resource 6b

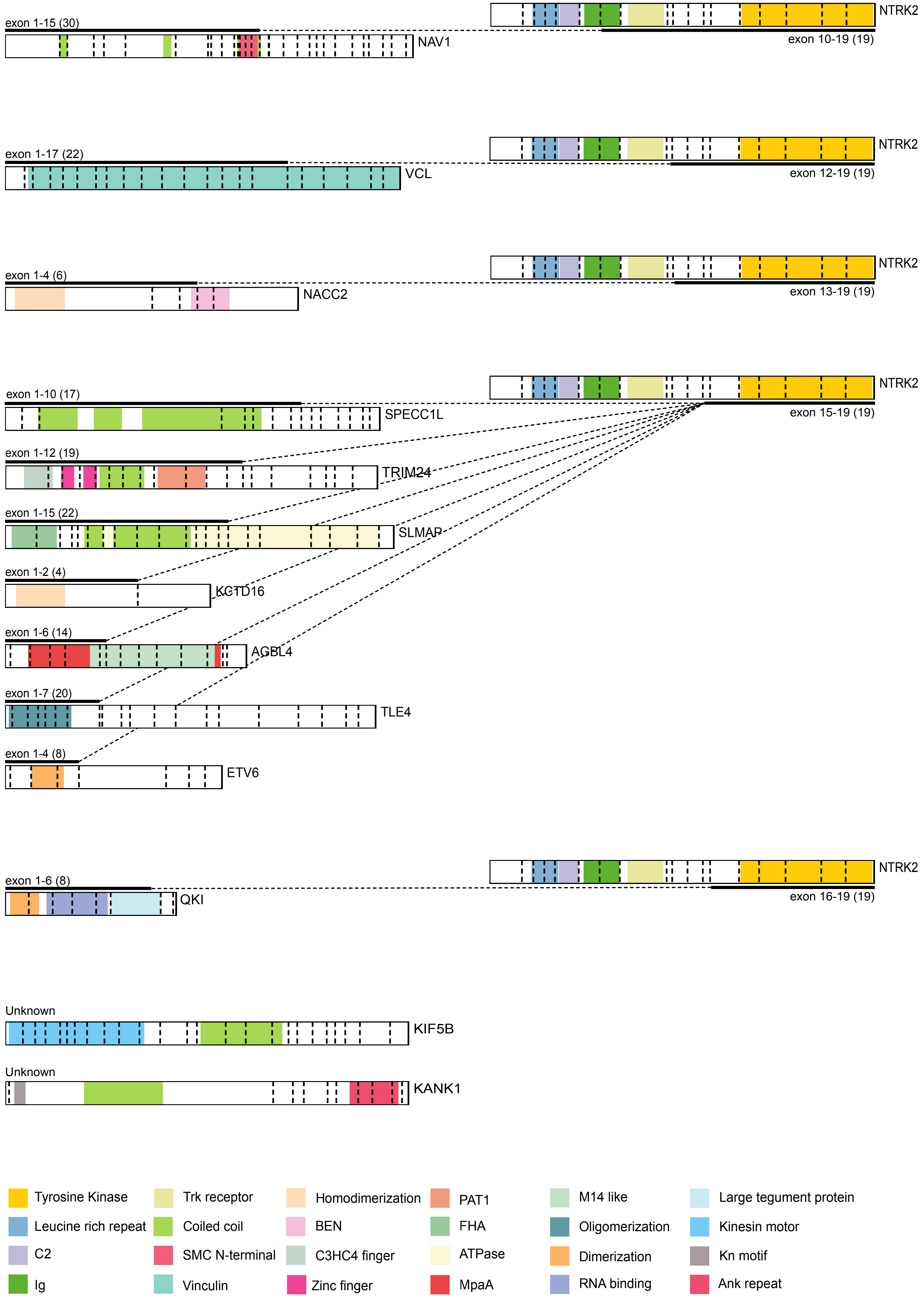

Online resource 6c

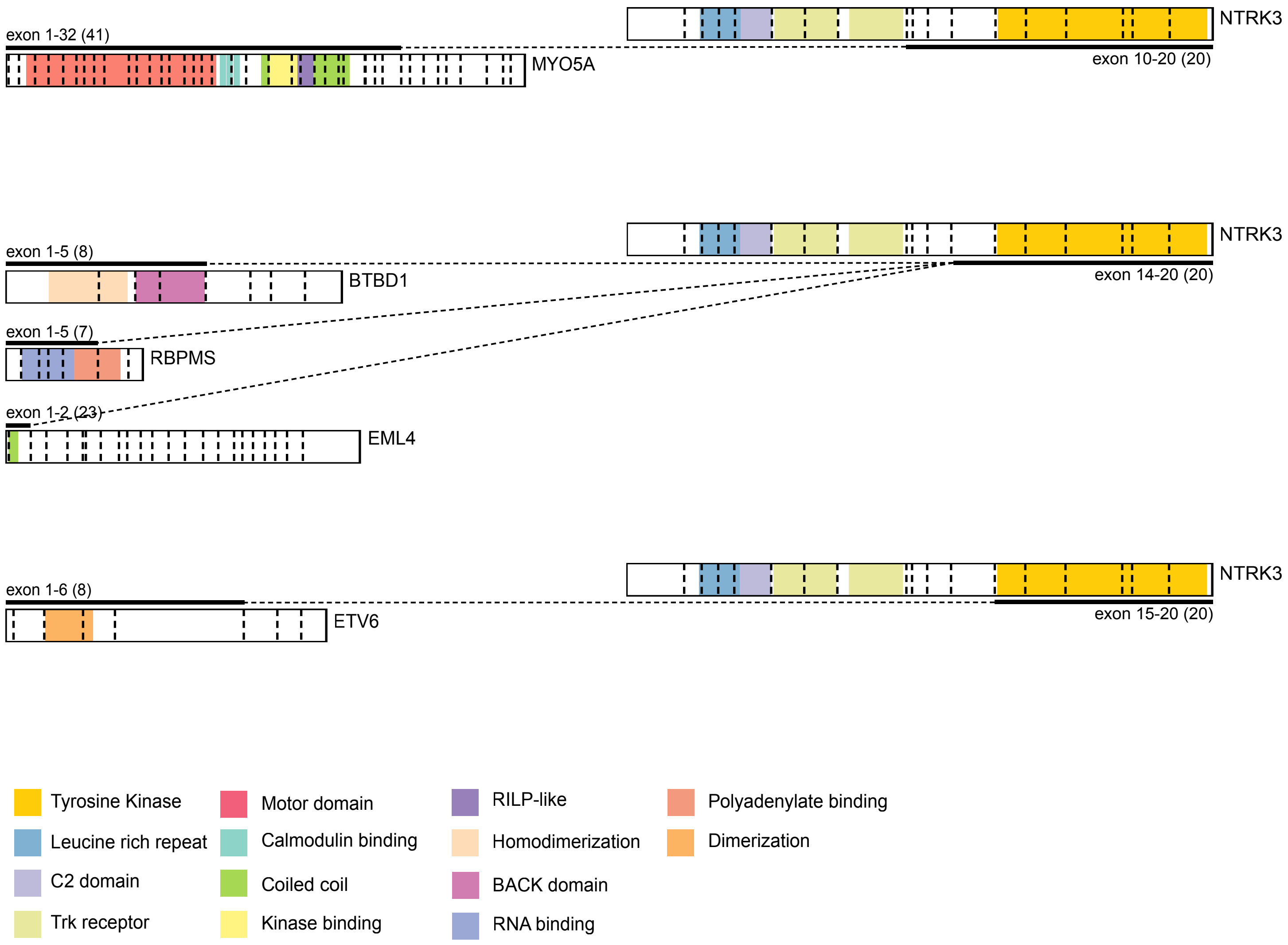

Online resource 7

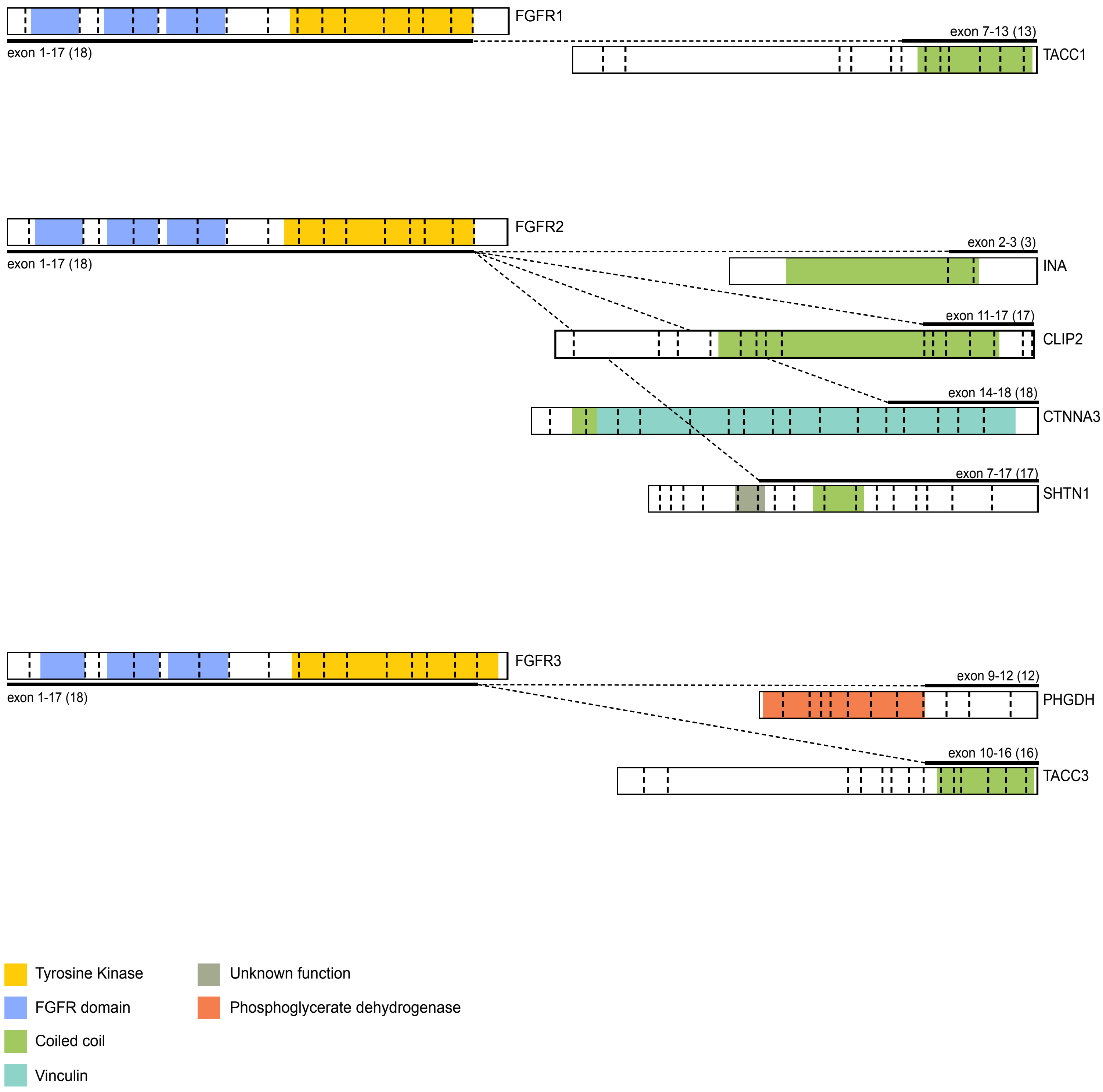

Online resource 8

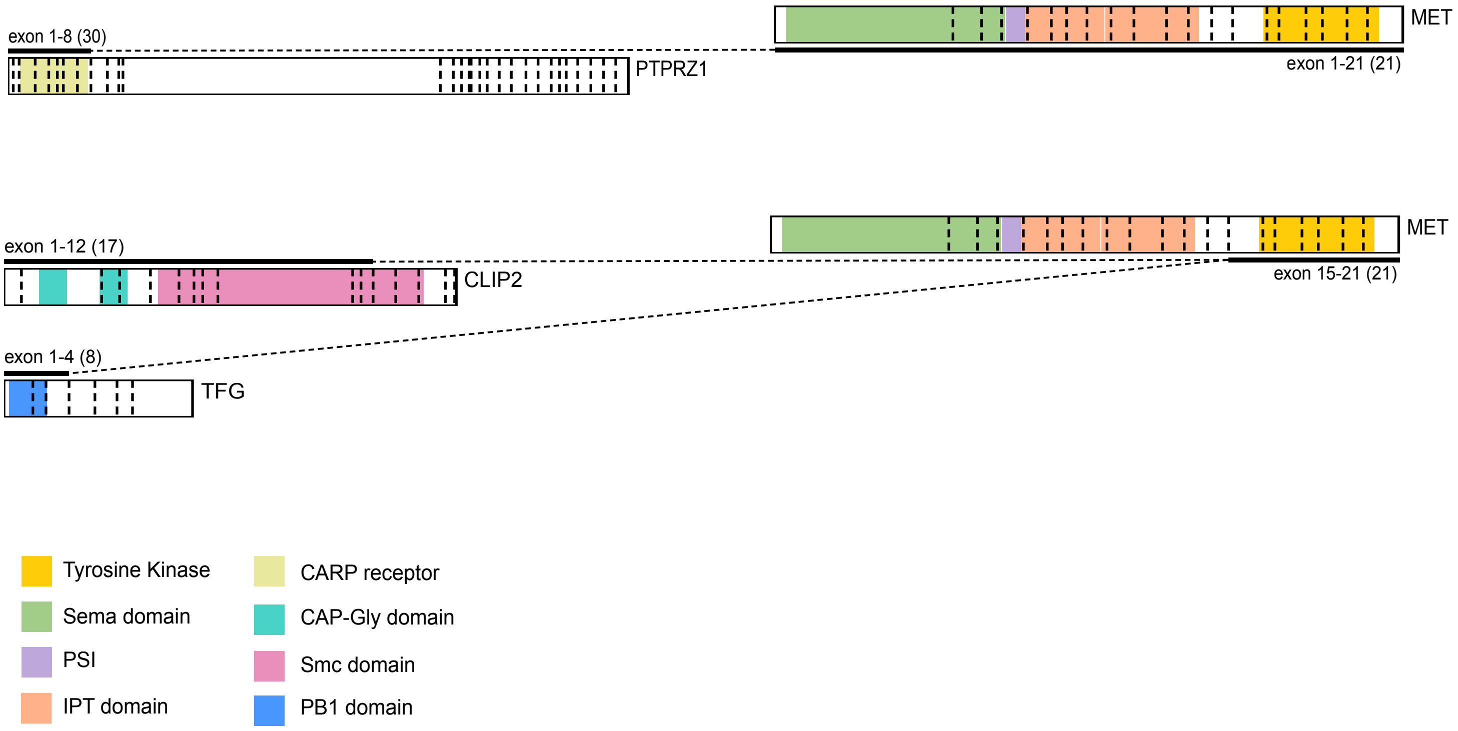

Online resource 9

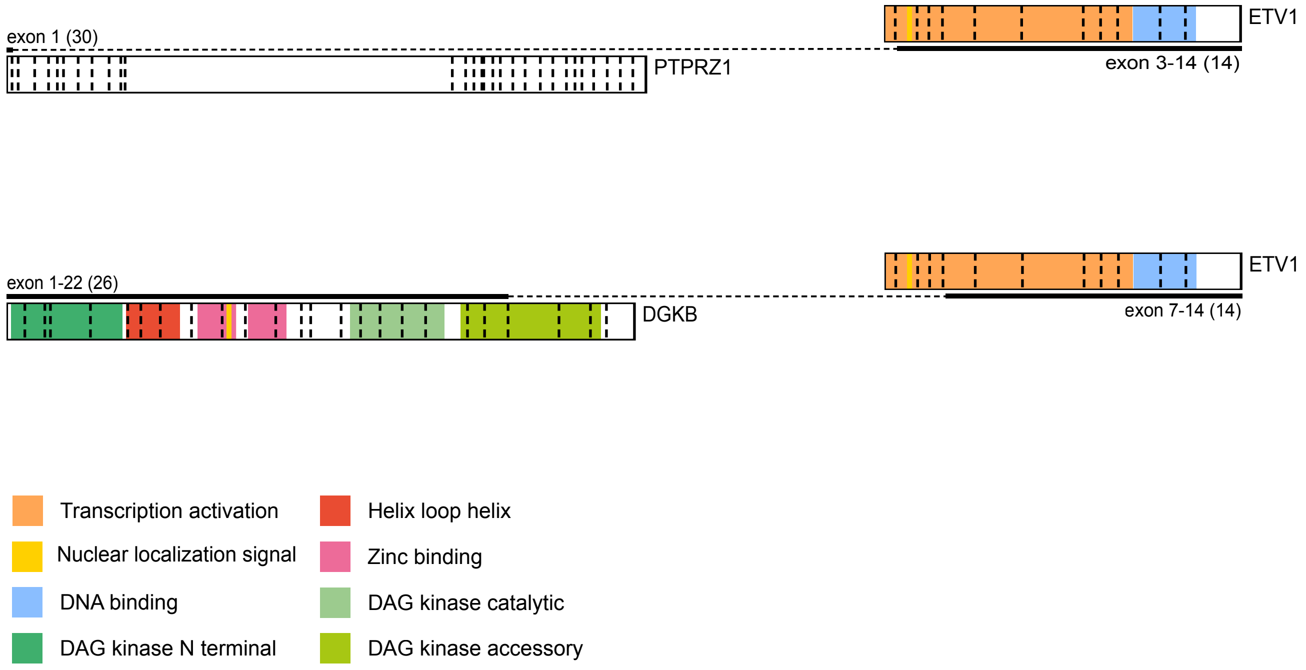

Online resource 10

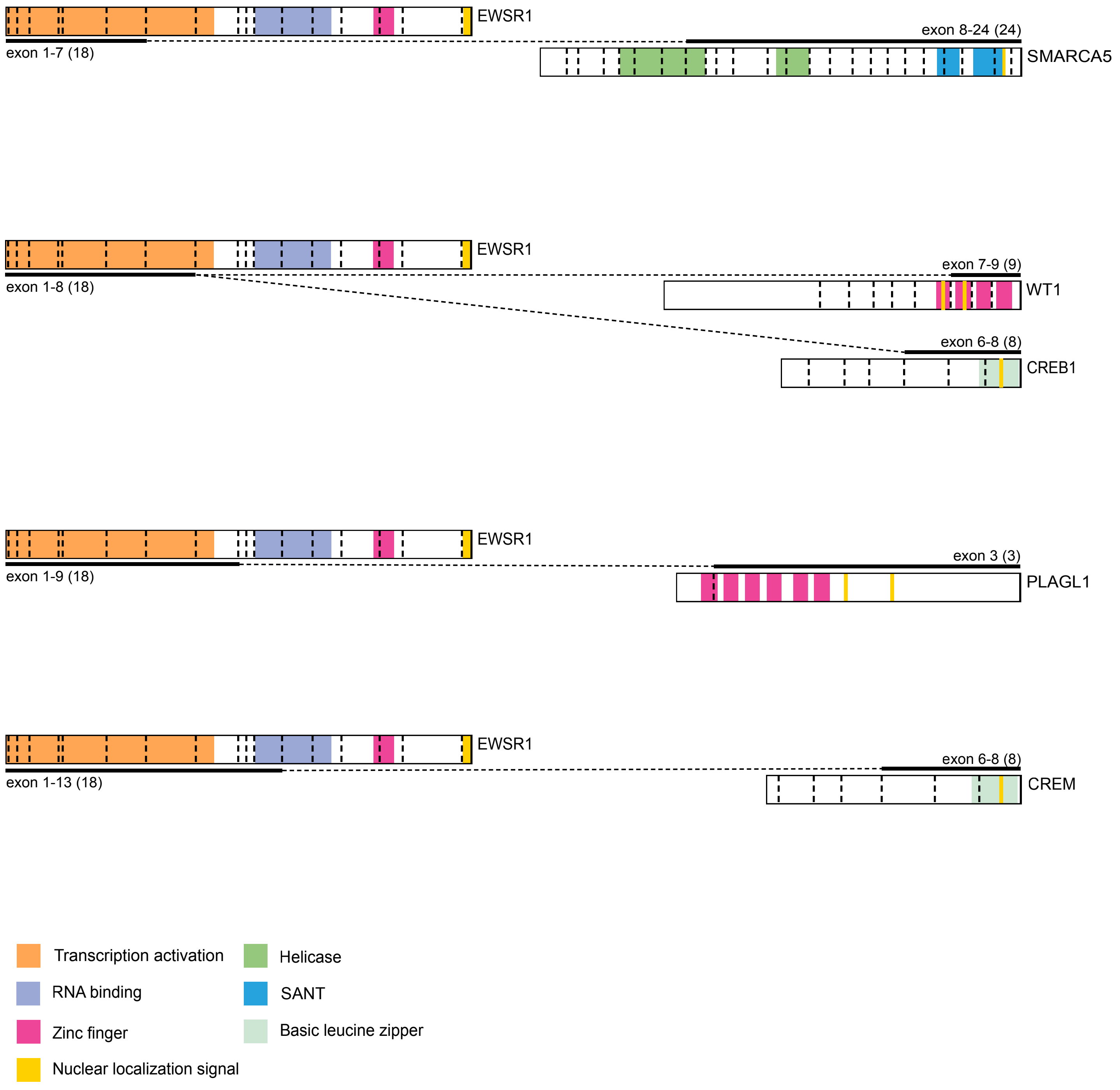

Online resource 11

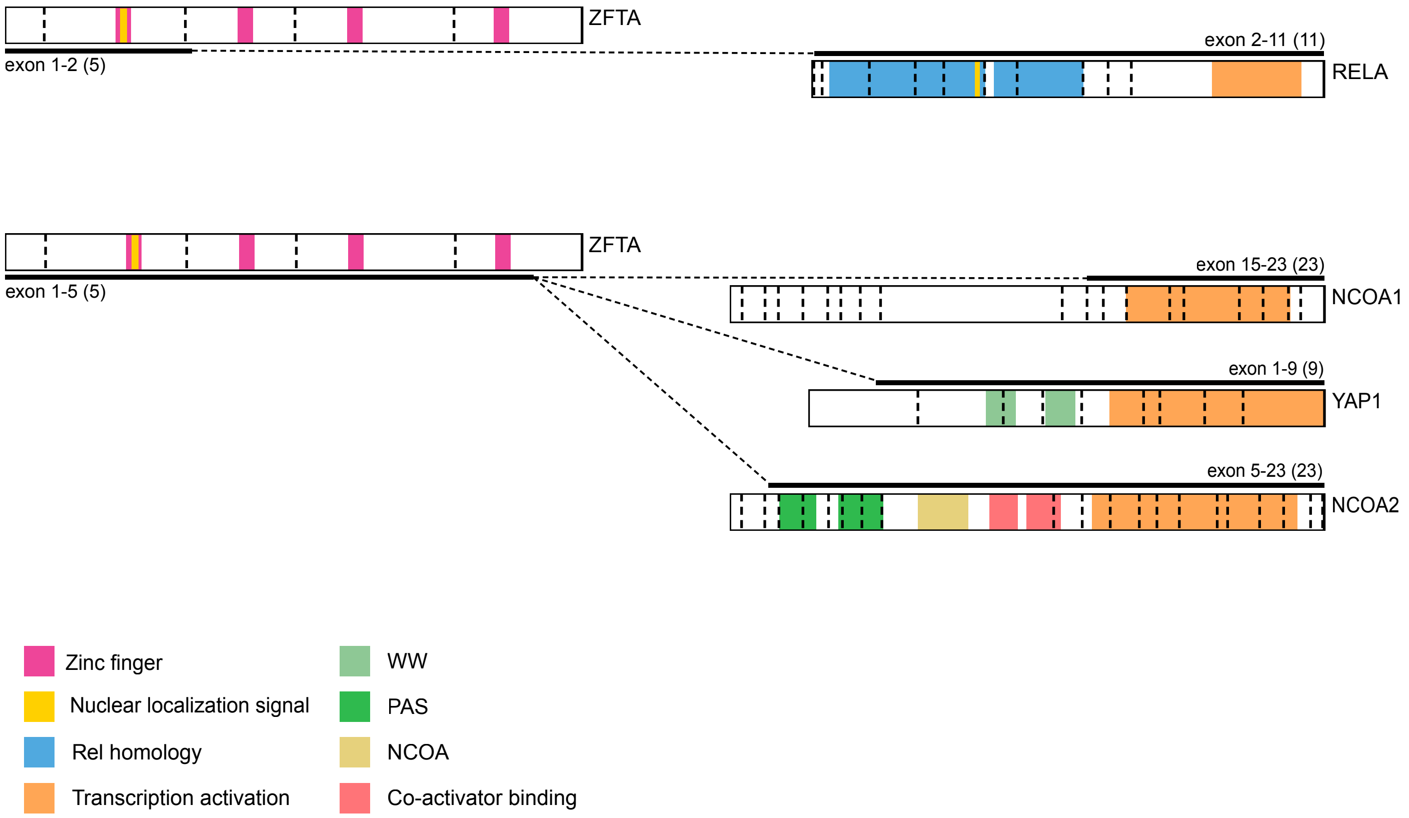

Online resource 12

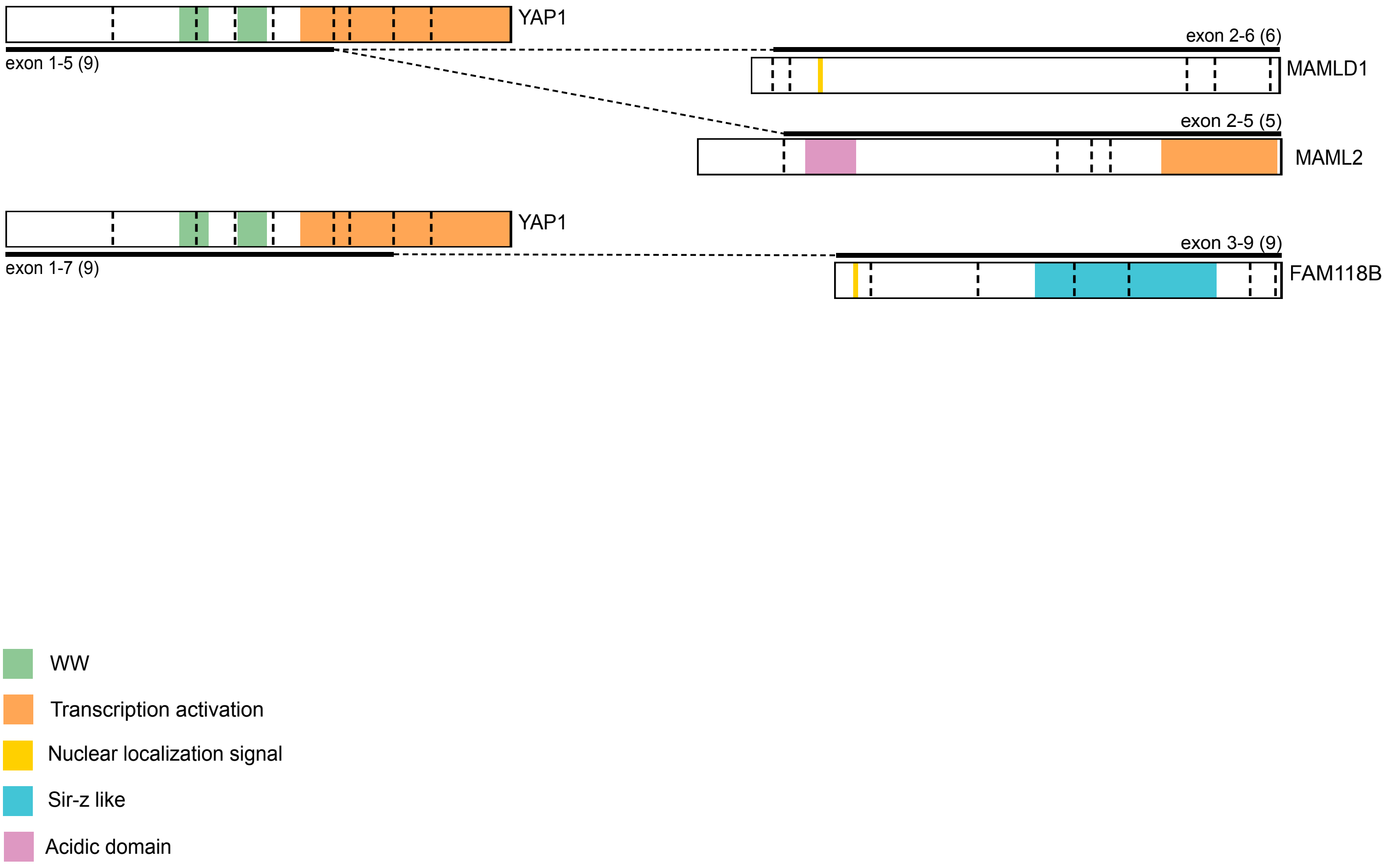

Online resource 13

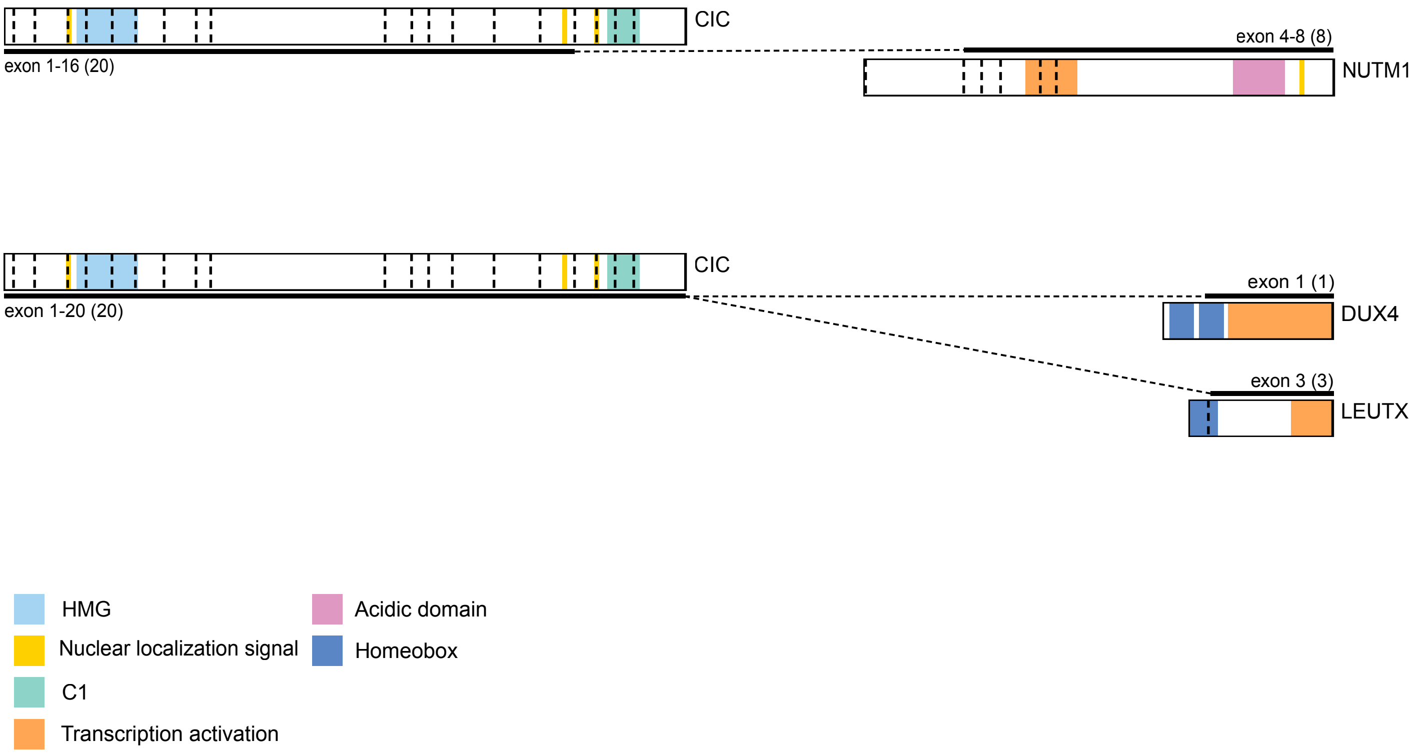

Online resource 14

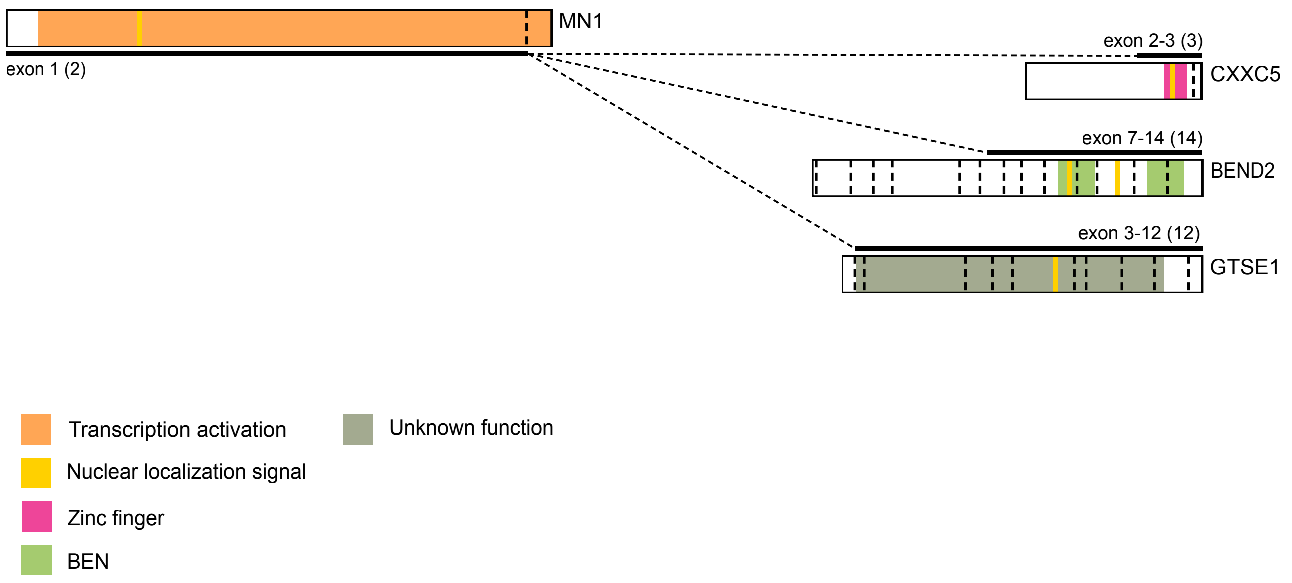

Online resource 15

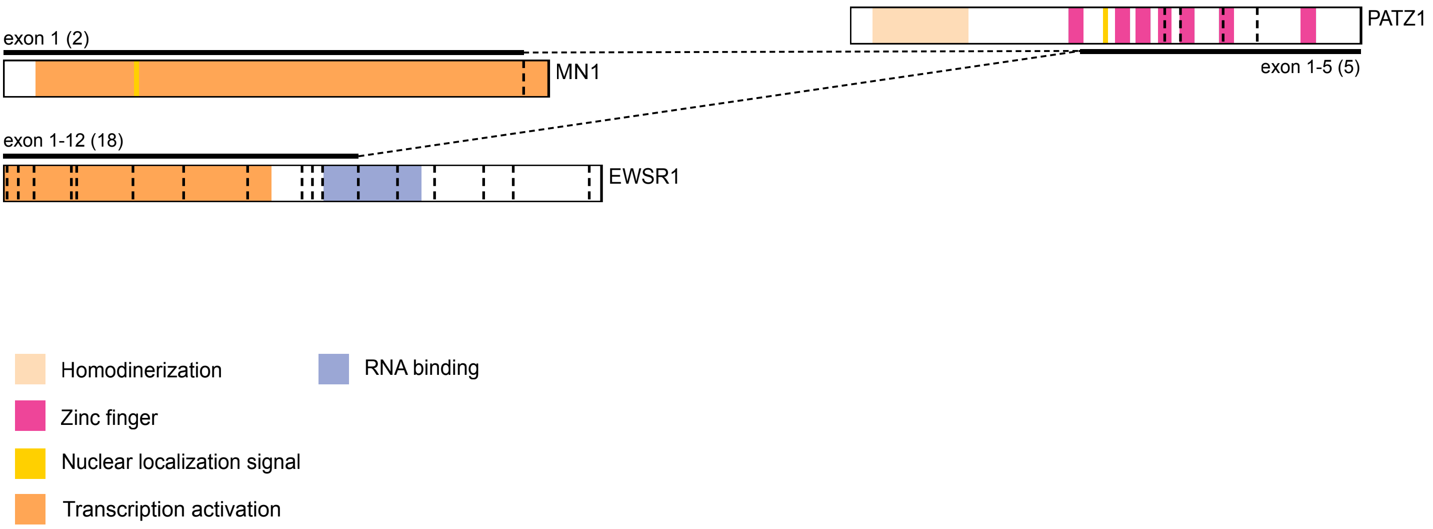

Online resource 16

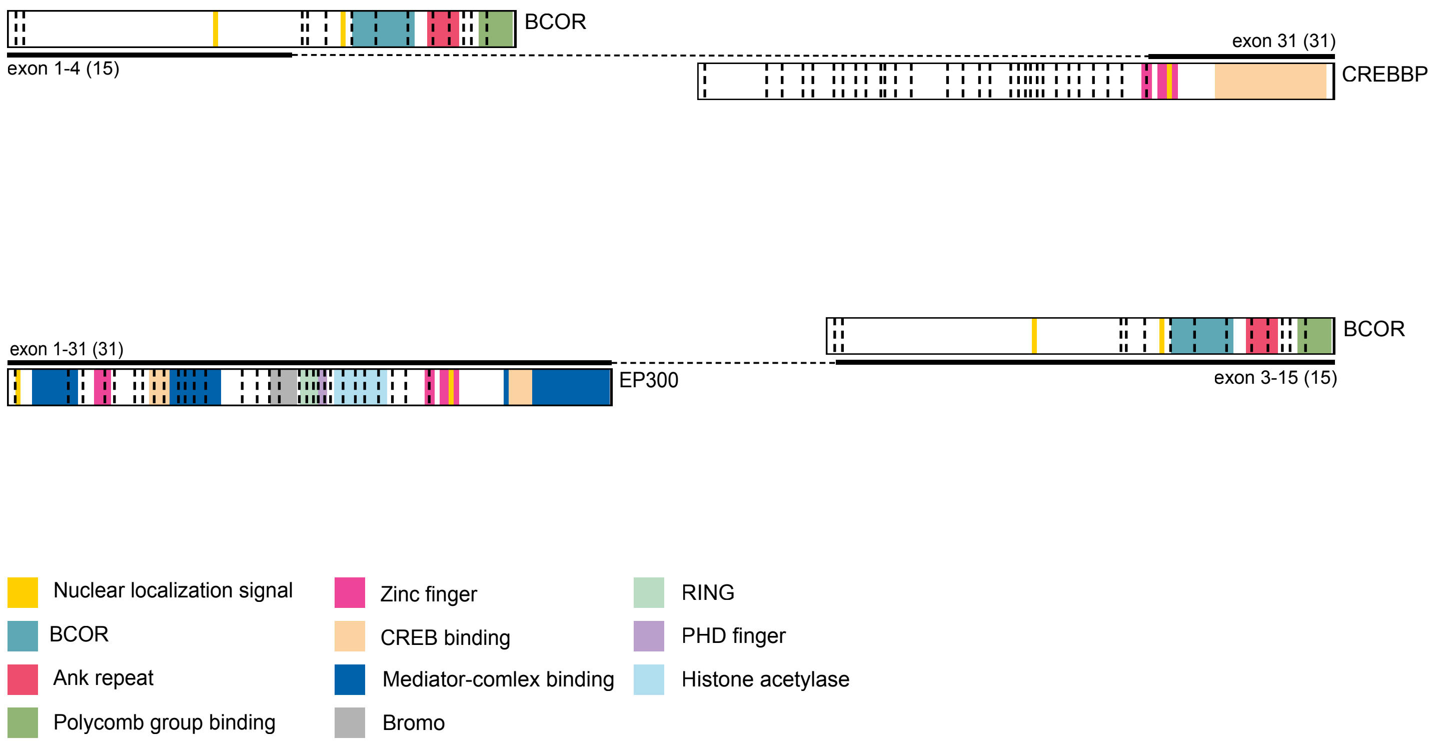

Online resource 17

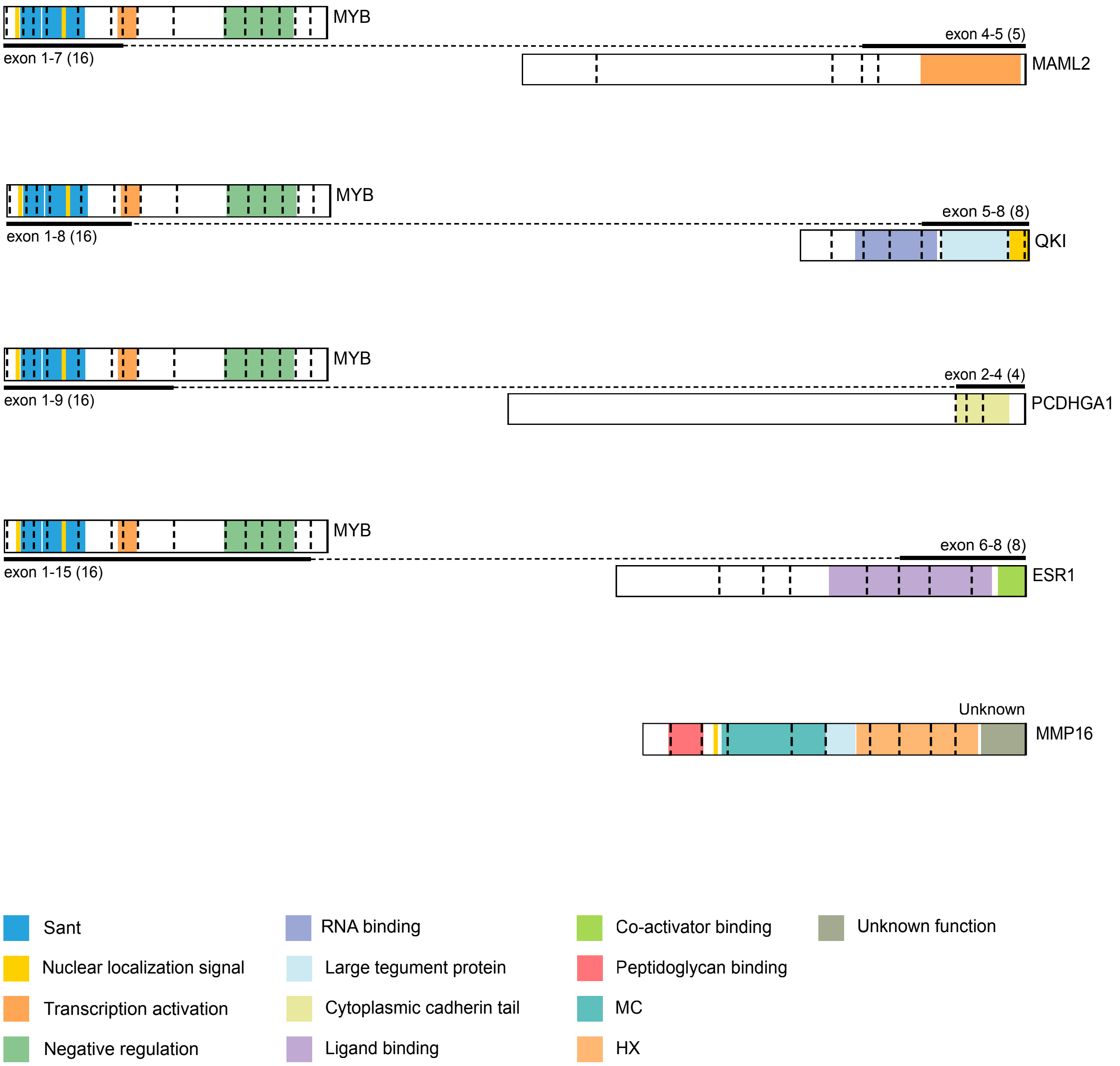

Online resource legends

Online resource 1 Table with all the fusion genes detected in pediatric CNS neoplasms.

Online resource 2 Fusions with BRAF. TRIM24 has no known breakpoint and is thus not connected. The exons shown are only the coding exons. For every protein, the exons that are retained in the fusion protein are specified, as well as the total exons (in between brackets) in the original protein. The most prominent breakpoint for each fusion is chosen, other breakpoints might also occur. Figure was generated with and adapted from ProteinPaint https://pecan.stjude. org/proteinpaint/.

Online resource 3 Fusions with RAF. TRIM 33 has no known breakpoints and is thus not connected. The exons shown are only the coding exons. For every protein, the exons that are retained in the fusion protein are specified, as well as the total exons (in between brackets) in the original protein. The most prominent breakpoint for each fusion is chosen, other breakpoints might also occur. Figure was generated with and adapted from ProteinPaint https://pecan.stjude. org/proteinpaint/.

Online resource 4 Fusions with ALK. MAP2 has no known breakpoints and is thus not connected. The exons shown are only the coding exons. For every protein, the exons that are retained in the fusion protein are specified, as well as the total exons (in between brackets) in the original protein. The most prominent breakpoint for each fusion is chosen, other breakpoints might also occur. Figure was generated with and adapted from ProteinPaint https://pecan.stjude. org/proteinpaint/.

Online resource 5 Fusions with ROS1. CHCHD3 has no known breakpoint and is thus not connected. The exons shown are only the coding exons. For every protein, the exons that are retained in the fusion protein are specified, as well as the total exons (in between brackets) in the original protein. The most prominent breakpoint for each fusion is chosen, other breakpoints might also occur. Figure was generated with and adapted from ProteinPaint https://pecan.stjude. org/proteinpaint/.

Online resource 6 Fusions with NTRK family members. The exons shown are only the coding exons. For every protein, the exons that are retained in the fusion protein are specified, as well as the total exons (in between brackets) in the original protein. The most prominent breakpoint for each fusion is chosen, other breakpoints might also occur. **a** Fusions with NTRK1. **b** Fusions with NTRK2, KIF5B and KANK1 have no known breakpoints and are thus not connected. **c** Fusions with NTRK3. Figure was generated with and adapted from ProteinPaint https://pecan.stjude. org/proteinpaint/.

Online resource 7 Fusions with FGFR. The exons shown are only the coding exons. For every protein, the exons that are retained in the fusion protein are specified, as well as the total exons (in between brackets) in the original protein. The most prominent breakpoint for each fusion is chosen, other breakpoints might also occur. Figure was generated with and adapted from ProteinPaint https://pecan.stjude. org/proteinpaint/.

Online resource 8 Fusions with MET. The exons shown are only the coding exons. For every protein, the exons that are retained in the fusion protein are specified, as well as the total exons (in between brackets) in the original protein. The most prominent breakpoint for each fusion is chosen, other breakpoints might also occur. Figure was generated with and adapted from ProteinPaint https://pecan.stjude. org/proteinpaint/.

Online resource 9 Fusions with ETV1. The exons shown are only the coding exons. For every protein, the exons that are retained in the fusion protein are specified, as well as the total exons (in between brackets) in the original protein. The most prominent breakpoint for each fusion is chosen, other breakpoints might also occur. Figure was generated with and adapted from ProteinPaint https://pecan.stjude. org/proteinpaint/.

Online resource 10 Fusions with EWSR1. The exons shown are only the coding exons. For every protein, the exons that are retained in the fusion protein are specified, as well as the total exons (in between brackets) in the original protein. The most prominent breakpoint for each fusion is chosen, other breakpoints might also occur. Figure was generated with and adapted from ProteinPaint https://pecan.stjude. org/proteinpaint/.

Online resource 11 Fusions with ZFTA. The exons shown are only the coding exons. For every protein, the exons that are retained in the fusion protein are specified, as well as the total exons (in between brackets) in the original protein. The most prominent breakpoint for each fusion is chosen, other breakpoints might also occur. Figure was generated with and adapted from ProteinPaint https://pecan.stjude. org/proteinpaint/.

Online resource 12 Fusions with YAP1. The exons shown are only the coding exons. For every protein, the exons that are retained in the fusion protein are specified, as well as the total exons (in between brackets) in the original protein. The most prominent breakpoint for each fusion is chosen, other breakpoints might also occur. Figure was generated with and adapted from ProteinPaint https://pecan.stjude. org/proteinpaint/.

Online resource 13 Fusions with CIC. The exons shown are only the coding exons. For every protein, the exons that are retained in the fusion protein are specified, as well as the total exons (in between brackets) in the original protein. The most prominent breakpoint for each fusion is chosen, other breakpoints might also occur. Figure was generated with and adapted from ProteinPaint https://pecan.stjude. org/proteinpaint/.

Online resource 14 Fusions with MN1. The exons shown are only the coding exons. For every protein, the exons that are retained in the fusion protein are specified, as well as the total exons (in between brackets) in the original protein. The most prominent breakpoint for each fusion is chosen, other breakpoints might also occur. Figure was generated with and adapted from ProteinPaint https://pecan.stjude. org/proteinpaint/.

Online resource 15 Fusions with PATZ1. The exons shown are only the coding exons. For every protein, the exons that are retained in the fusion protein are specified, as well as the total exons (in between brackets) in the original protein. The most prominent breakpoint for each fusion is chosen, other breakpoints might also occur. Figure was generated with and adapted from ProteinPaint https://pecan.stjude. org/proteinpaint/.

Online resource 16 Fusions with BCOR. The exons shown are only the coding exons. For every protein, the exons that are retained in the fusion protein are specified, as well as the total exons (in between brackets) in the original protein. The most prominent breakpoint for each fusion is chosen, other breakpoints might also occur. Figure was generated with and adapted from ProteinPaint https://pecan.stjude. org/proteinpaint/.

Online resource 17 Fusions with MYB. The exons shown are only the coding exons. For every protein, the exons that are retained in the fusion protein are specified, as well as the total exons (in between brackets) in the original protein. The most prominent breakpoint for each fusion is chosen, other breakpoints might also occur. Figure was generated with and adapted from ProteinPaint https://pecan.stjude. org/proteinpaint/.
